# Identification of rare microbial colonizers of plastic materials incubated in a coral reef environment

**DOI:** 10.3389/fmicb.2023.1259014

**Published:** 2023-10-05

**Authors:** Sebastian L. Singleton, Edward W. Davis, Holly K. Arnold, An Mei Y. Daniels, Susanne M. Brander, Rachel J. Parsons, Thomas J. Sharpton, Stephen J. Giovannoni

**Affiliations:** ^1^Department of Microbiology, Oregon State University, Corvallis, OR, United States; ^2^Bermuda Institute of Ocean Sciences, St. George's, Bermuda; ^3^Department of Fisheries, Wildlife, and Conservation Sciences, Coastal Oregon Marine Experiment Station, Oregon State University, Newport, OR, United States

**Keywords:** plastisphere, microbiome, polyolefins, microbial succession, *in situ*, 16S rDNA, FTIR, HIM

## Abstract

Plastic waste accumulation in marine environments has complex, unintended impacts on ecology that cross levels of community organization. To measure succession in polyolefin-colonizing marine bacterial communities, an *in situ* time-series experiment was conducted in the oligotrophic coastal waters of the Bermuda Platform. Our goals were to identify polyolefin colonizing taxa and isolate bacterial cultures for future studies of the biochemistry of microbe-plastic interactions. HDPE, LDPE, PP, and glass coupons were incubated in surface seawater for 11 weeks and sampled at two-week intervals. 16S rDNA sequencing and ATR-FTIR/HIM were used to assess biofilm community structure and chemical changes in polymer surfaces. The dominant colonizing taxa were previously reported cosmopolitan colonizers of surfaces in marine environments, which were highly similar among the different plastic types. However, significant differences in rare community composition were observed between plastic types, potentially indicating specific interactions based on surface chemistry. Unexpectedly, a major transition in community composition occurred in all material treatments between days 42 and 56 (*p* < 0.01). Before the transition, Alteromonadaceae, Marinomonadaceae, Saccharospirillaceae, Vibrionaceae, Thalassospiraceae, and Flavobacteriaceae were the dominant colonizers. Following the transition, the relative abundance of these taxa declined, while Hyphomonadaceae, Rhodobacteraceae and Saprospiraceae increased. Over the course of the incubation, 8,641 colonizing taxa were observed, of which 25 were significantly enriched on specific polyolefins. Seven enriched taxa from families known to include hydrocarbon degraders (Hyphomonadaceae, Parvularculaceae and Rhodobacteraceae) and one n-alkane degrader (*Ketobacter* sp.). The ASVs that exhibited associations with specific polyolefins are targets of ongoing investigations aimed at retrieving plastic-degrading microbes in culture.

## Introduction

The Anthropocene epoch is characterized by post-industrial revolution human activity including the widespread accumulation of plastic material in natural systems. Plastics and other anthropogenic artifacts in stratigraphic layers are the definitive markers of the Anthropocene (Zalasiewicz et al., 2017). This period can be referred to as the rise of plastic reliance, wherein the production of plastic polymers and subsequent sequestration in terrestrial and aquatic environments is ubiquitously observed worldwide (Barnes et al., 2009; Pathak and Nichter, 2019; Dudek et al., 2020; Wright et al., 2020). The influx of plastics into the natural environment is an unintentional ramification of under-developed waste management systems supporting the load of a global society dependent on low-cost, recalcitrant materials (Barnes et al., 2009; Pathak and Nichter, 2019). Once plastic wastes enter natural habitats, interactions with various abiotic factors (UV irradiation, thermal degradation, mechanical abrasion) weather, fragment and distribute them throughout overlapping habitats and ecosystem trophic levels (Amaral-Zettler et al., 2020; Wright et al., 2020).

Characterized as thermoplastics, polyethylene (PE) and polypropylene (PP) are polyolefin petroleum-based compounds that comprise most of the plastic produced annually (Plastics Europe, 2020; Plastics Europe, 2021; Plastics Europe, 2022). A meta-analysis of international marine plastic investigations by Erni-Cassola et al. (2019), revealed that PE, PP, and polyamide resins (PA) are the most abundant plastics in surface marine systems (23, 13, and 20%, respectively). Plastic Europe reported that of the estimated 390.7 Mt. of plastics globally produced in 2021, the proportion of polyolefin polymers was 19.3% PP, 14.4% LDPE/LLDPE and 12.5% HDPE/MDPE (Plastics Europe, 2022). As an indirect result of the current rate of production, plastics have become prominent in the surface marine environment from decades of mismanagement, unintended anthropogenic actions, and the polymer’s low-density relative to seawater (Donlan, 2002; Pathak and Nichter, 2019; Wright et al., 2020; Cheng et al., 2021; Villarrubia-Gómez et al., 2023).

In marine environments, plastic polymers reside for extensive periods of time, serving to pollute the environment and interrupt ecosystem interactions (Thompson et al., 2009; Liu et al., 2019). The term “plastisphere” describes plastic-associated organismal (micro and macro) communities and their ecological interactions (Zettler et al., 2013; Liu et al., 2019; Wright et al., 2020). Prokaryotes, largely within the phyla Proteobacteria and Bacteroidota, and eukaryotic microbes ranging from larval macrofauna (such as bryozoans and barnacles) to microfauna (diatoms, rotifers, brown algae) commonly colonize plastic substrates (Wright et al., 2020; Latva et al., 2022). The plastisphere encompasses a variety of ecological niches that includes phototrophs (e.g., phytoplankton), saprotrophs (e.g., marine fungi), symbionts (e.g., sulfide-oxidizers associated with eukaryotic organisms), heterotrophs (e.g., bryozoan larvae and hydrocarbonoclastic bacteria) and predators (Debroas et al., 2017; Amaral-Zettler et al., 2020; Wright et al., 2020). Community composition within the plastisphere is driven by a complex assortment of factors, such as polymer type, surface properties (% crystallinity), size, sunlight, and seawater parameters (salinity, carbonate, pH) (Oberbeckmann et al., 2018; Xu et al., 2019; Amaral-Zettler et al., 2020; Harvey et al., 2020; Wright et al., 2020). Of all factors, spatial location and seasonality have been observed to have the greatest impact on community composition (Oberbeckmann et al., 2014, 2016; Jacquin et al., 2019; Amaral-Zettler et al., 2020; Wright et al., 2020).

Amid future projections of increased plastic production (Plastics Europe, 2020; Plastics Europe, 2021; Plastics Europe, 2022; Ackerman and Levin, 2023), hope that these materials will not persist for eons comes from repeated reports of a growing number of microbial taxa capable of plastic colonization and biodegradation (Hadar and Sivan, 2004; Sudhakar et al., 2008; Balasubramanian et al., 2010; Harshvardhan and Jha, 2013; Santo et al., 2013; Yoshida et al., 2016; Paço et al., 2017; Syranidou et al., 2017; Auta et al., 2018; Mohanrasu et al., 2018; Devi et al., 2019; Oberbeckmann and Labrenz, 2019; Roager and Sonnenschein, 2019; Dey et al., 2020; Elumalai et al., 2021; Gao and Sun, 2021). Within the marine system, a handful bacteria have been observed to degrade high molecular weight polyolefin polymers: *Kocuria palustris*, *Brevibacillus borstelensis, Bacillus pumilis*, *B. subtilis, B. cereus, B. sphericus, Exiguobacterium* sp., *Halomonas* sp., *Ochrobactrum* sp., *Rhodococcus* sp.*, Arthrobacter* sp., *Pseudomonas* spp., and *Bacillus* spp. strains (Sudhakar et al., 2008; Balasubramanian et al., 2010; Auta et al., 2018; Mohanrasu et al., 2018; Devi et al., 2019; Elumalai et al., 2021; Gao and Sun, 2021). Biodegradation by marine bacteria is influenced by multiple factors: time, temperature, salinity, pH, and nutrients in the environment (Debroas et al., 2017; Tu et al., 2020; Wright et al., 2020). In the marine system reports of polyolefin specific surface degradation rate (defined as the volume lost by removal of a layer of thickness in a specified time) without fillers or degradation accelerating treatments range from 4.3 to 15 μm year^−1^ (HDPE and LDPE respectively). When observed across all common polymers (PET, HDPE, PVC, LDPE, PP, and PS) the averaged rate is ~10 μm year^−1^ (Chamas et al., 2020). As alluded to above, the rate of degradation can vary depending on the bacterial strain and environmental conditions (Roager and Sonnenschein, 2019; Tu et al., 2020; Wright et al., 2020). Additionally, polymers with high crystalline fractions (e.g., HDPE) are typically associated with lower degradation rates, due to microbes preferentially targeting the less organized amorphous fractions, which possess no predictable order (Sudhakar et al., 2008; Chamas et al., 2020).

Research on microbial colonization of plastics is motivated by hope that more taxa capable of degrading these materials, and conditions that stimulate the degradative process, will be found. In this study we hypothesize that plastic degrading taxa might be relatively slow growing, late colonizers of plastic materials that that colonize the relatively small biofilm niche at the physical surface of the substrate. While we have learned much in recent years about microbial colonization of plastic surfaces over short time scales, especially in marine ecosystems, relatively little is known about the long-term dynamics of plastic-associated microbial communities (Oberbeckmann et al., 2016, 2018; Dudek et al., 2020; Erni-Cassola et al., 2020; Pinto et al., 2020; Kumar et al., 2022; Latva et al., 2022). Studies that examine later stages of plastic surface biofilm development *in situ* are uncommon (Sudhakar et al., 2007; De Tender et al., 2017; Tu et al., 2020; Wallbank et al., 2022). Investigations of plastic-attached community assemblages at later stages of colonization are needed to improve understanding of plastisphere community succession and to identify slow-growing taxa capable of polyolefin degradation. In the summer of 2021, we deployed three of the most prevalent plastics found in the surface ocean (HDPE, LDPE, and PP) into the oligotrophic waters of the Bermuda Platform for 11 weeks to address this knowledge gap. Unexpectedly, a hurricane developed mid-experiment that correlated with a stark transition in the plastic communities. Within the biofilms we identified many abundant common cosmopolitan colonizers (>5%) as well as rare taxa associated with specific polymer types (≤1%), some of which were previously identified plastic-degraders, and others of which had not previously been associated with plastics. These ASVs are candidates for future studies aimed at expanding knowledge of microbial plastics degradation.

## Materials and methods

### Sampling design, processing and collection

**Figure 1:**
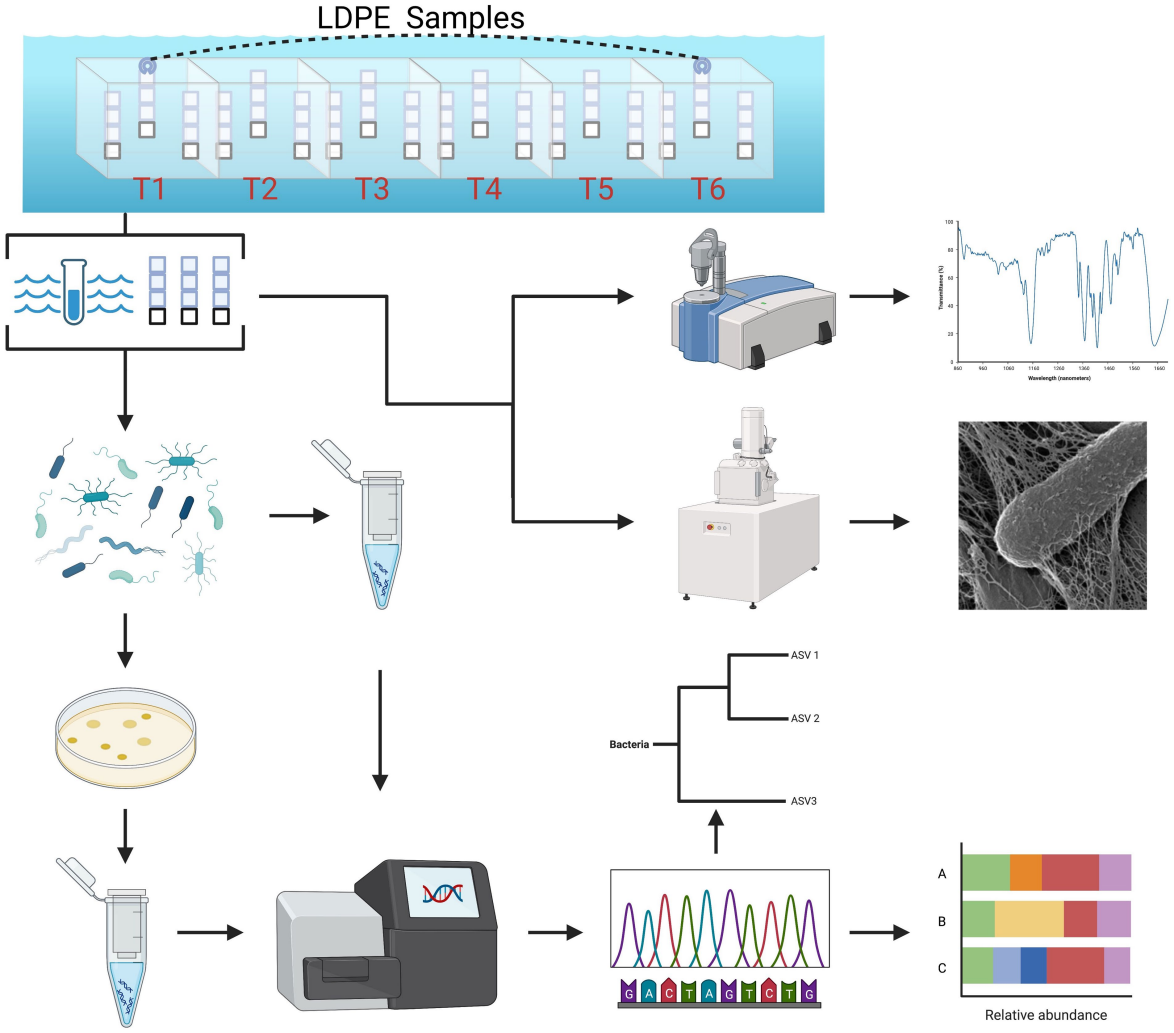
Summarized experimental workflow: sample collection (biweekly over 3 months) to downstream analysis [cultivation, 16S (V4) sequence analysis, ATR-FTIR spectral analysis and HIM imaging].

This longitudinal marine polyolefin *in situ* exposure study was conducted from July to September 2021 in the coastal waters of the Bermuda Platform. Located in Ferry Reach, the study site was adjacent to the small boat dock of the Bermuda Institute of Ocean Sciences (32.369799, −64.696481). Three polyolefin polymer types without color additives were selected for marine incubation (United States Plastic Corporation, Lima, OH): high-density polyethylene (HDPE), low-density polyethylene (LDPE) and polypropylene (PP). Prior to incubation, each polymer type was cut into 40 × 40 × 1.5 mm coupons and thoroughly rinsed with filtered deionized water, followed by 70% ethanol in triplicate. The coupons were separated by polymer type and incubation groups (2, 4, 6, 8, 10, 12 weeks), strung together with fishing line in chains of 3 and suspended inside semi-transparent polypropylene crates with 20–30 25 mm holes per side (Figure 1). As an inert material control for the assessment of cell growth (Supplementary Figure S1) and community compositions on the plastics, 3 glass coupons were added to each crate. To avoid colonization by larval macrofauna, each crate was covered in 75 μm Nitex mesh. Crates were assigned by polymer type and sample event; 18 crates were assembled in total. The individual crates were rigged together by polymer type in series and deployed ~30 cm below the water surface attached to a boat moor.

Over the incubation period, 6 time points were carried out in 2-week intervals to collect and extract bacteria from the ambient seawater and the coupons (plastic/glass). Every time point, including the day of deployment, 1 L of ambient seawater was collected on 0.1 μm Sterivex pressure filters (Millipore-Sigma) using a peristaltic pump. Due to in-clement weather caused by the emergence of Tropical Storm Henri, the incubation period was shortened by a week and collected on day 77 to avoid sample loss. Following sample collection, the coupons were placed in 0.1 μm filtered seawater on ice and immediately transported to the Parsons Microbial Ecology Lab at BIOS for processing. The cells were dislodged from the plastic coupon using a generic oral irrigator precleaned with 80% ethanol and filtered (0.1 μm) seawater and then filled with filtered (0.1 μm) sterile seawater on the lowest setting. The dislodged cells suspensions were equally divided into aliquots for prokaryotic abundance, cultivation, and DNA extraction. Additionally, a subset was left unaltered and preserved in 5% glutaraldehyde for high resolution helium ion microscopy (HIM). Whereas cell dislodged coupons were retained for surface change characterization (ATR-FTIR).

### Assessment of prokaryotic abundance

DAPI stain cell enumeration: At every timepoint, dislodged cell suspension samples from each material type (three biological replicates) were fixed with 10% formalin, loaded (2 mL) onto 0.2 μm polycarbonate filters pre-stained with Irgalan Black (0.2 g in 100 mL 2% acetic acid) and stained with 0.5 mL of 4′,6-diamidino-2-phenylindole dihydrochloride (DAPI; Sigma-Aldrich, St. Louis, MO, United States; 5 μg mL^−1^) for 3 min (Porter and Feig, 1980). Ultraviolet epifluorescence microscopy per sample at a magnification of x1000.

### Cultivation of polyolefin-colonizing bacteria

Cultivation was performed on 0.3% w/v polyolefin-enriched low-nutrient heterotrophic solid marine media. The cell suspensions were plated onto different plates based on the polymer type from which they were collected and incubated at 30°C in the dark until colonies formed (1–4 weeks). Further description of the cultures yielded from this investigation, media component details and an assessment of polyolefin biodegradation capabilities will be thoroughly discussed in a subsequent manuscript.

### Documentation of microbe-plastic interface: HIM imaging

**Figure 2:**
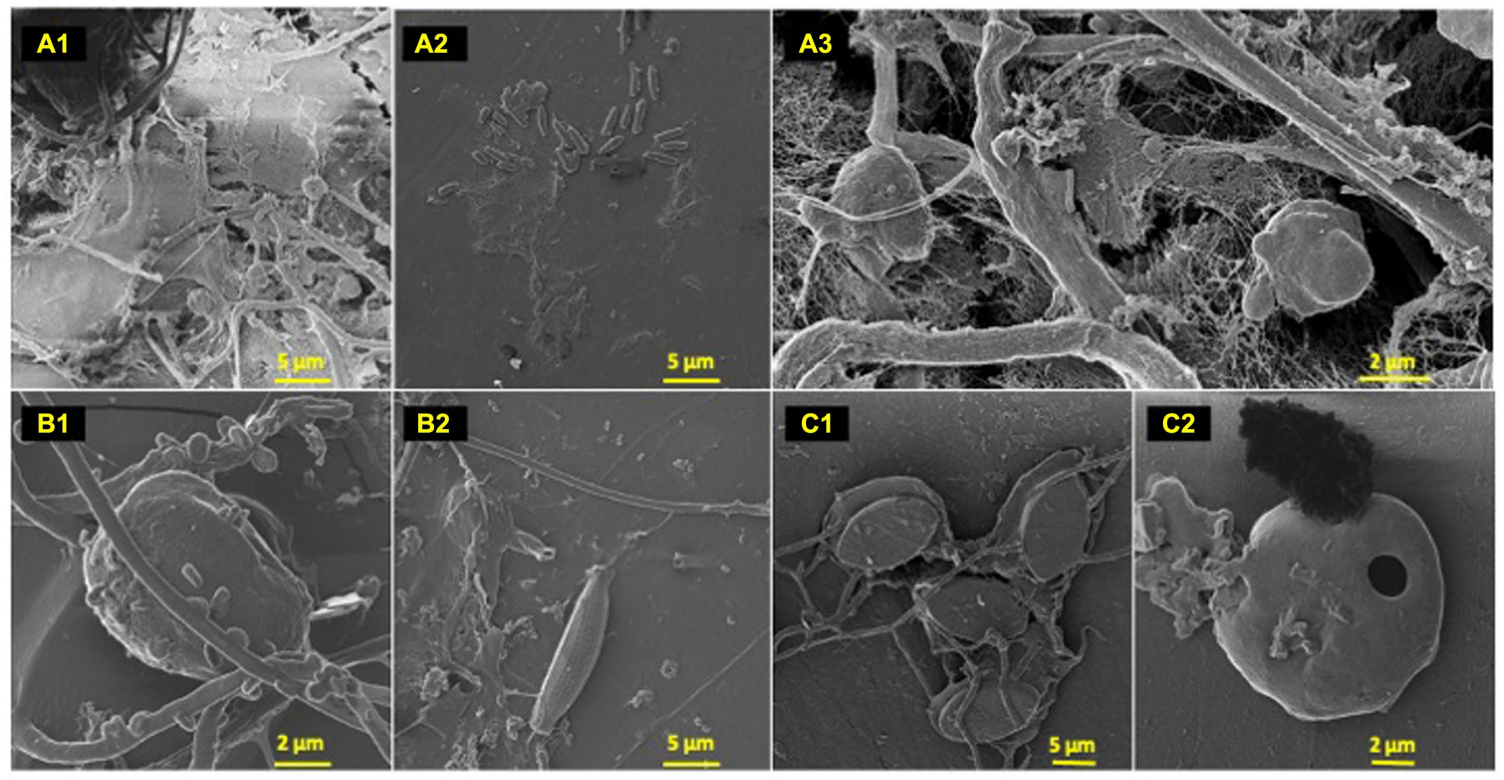
Polyolefin colonizers. HIM images of polyolefin colonizing species post 77-day marine incubation. HDPE: **(A)** (1–3); LDPE: **(B)** (1–2); PP: **(C)** (1–2).

**Figure 3:**
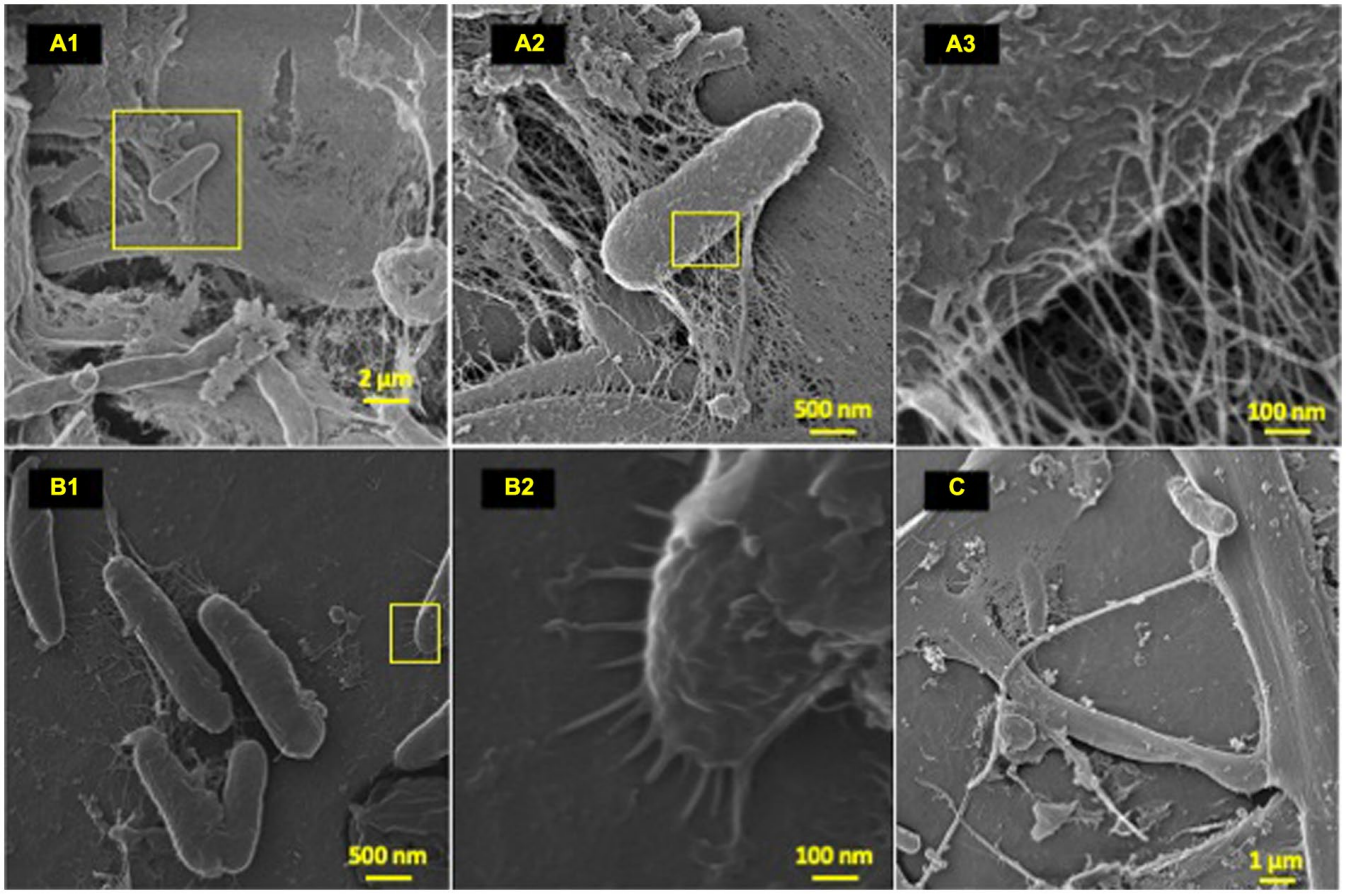
Microbial attachment phenotypes. HIM images of attachment phenotypes utilized by HDPE colonizing species post-77-day marine incubation. **(A–C)** Highlight various attachment and cell–cell interaction phenotypes; **(A)** (1–3): EPS formation from a bacilli; **(B)** (1–2): polymer-cell adhesion via bacilli pili projections; **(C)** intricate multi cell–cell interaction through formation of single thick (~200 nm) bifurcating pili, which spans ~10 μm at furthest point.

**Figure 4:**
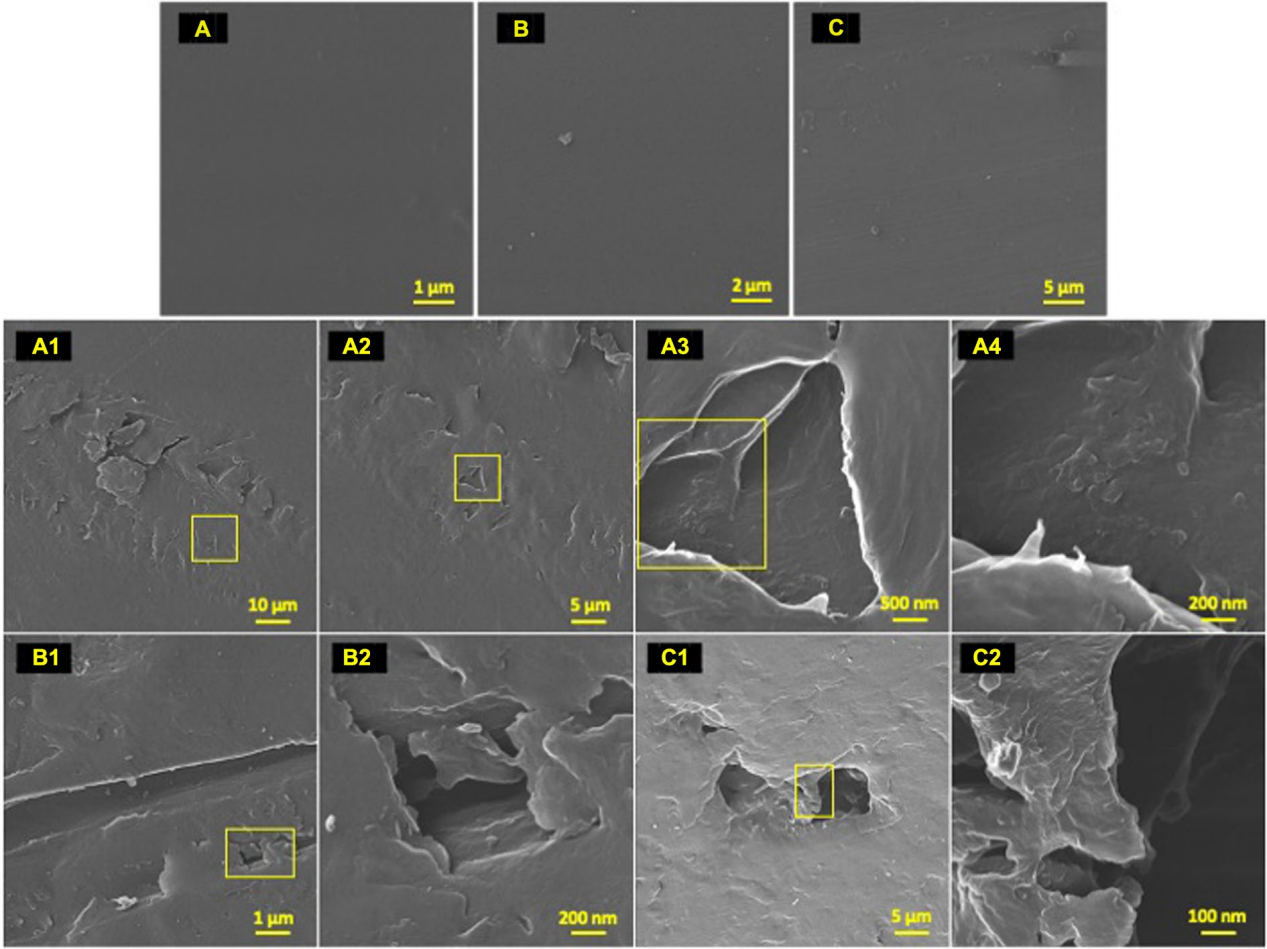
Post incubation biodegradation artifacts. HIM images of 77-day incubated polyolefins with biofilm removed in contrast to unexposed controls to exhibit artifacts of biodegradation by colonizing taxa. Marine-incubated LDPE **(A)** (1–4), HDPE **(B)** (1–2) and PP **(C)** (1–2). Unexposed polyolefins: LDPE **(A)**, HDPE **(B)** and PP **(C)**.

To characterize polyethylene and polypropylene colonizing bacterial taxa present at the termination of the experiment, high-resolution images (200 μm to <100 nm) were captured using HIM (Figures 2–4). Following the 77-day incubation, the colonized plastic coupons were collected, immediately placed into 0.1 μm filtered seawater fixed with 5% glutaraldehyde, stored at 4^o^ C and remained until processing was performed ~1.5 years later. Evidence by Stolz and Margulis (1984) and Giovannoni (unpublished) support the use of 2.5–5% glutaraldehyde for the fixation of biological samples and subsequent preservation with no deleterious effects on surface characteristics for years to indefinitely, if kept at 4–8°C. A day prior to critical point drying (CPD), the samples were removed from the fixative and separated into two groups: 77-day (exposed) and 77-day (digested). The exposed samples were placed into Swinnex 13 mm filter holders (Millipore-Sigma; St. Louis, Missouri, United States), washed of marine salts in series 0.1um filtered seawater (FSW) with 18.2 MΩ·cm filtered DI water (Milli-Q Water Purification System; Millipore Sigma; 75, 50, 25, 0% FSW for 5 min each), desiccated in series (10, 25, 50, 75% ethanol for 10 min each) and placed in 100% ethanol for 24 h. The digested samples were incubated in 0.2 μm filtered 10% KOH for 48 h on a shake plate (80 rpm) to digest attached organic matter. The digested samples and unexposed controls were rinsed 3x in sterile deionized water, 3x in 70% ethanol and air dried overnight in a clean chemical fume hood. The samples were critical point dried in liquid CO_2_ using an Tousimis Autosamdri-815 CPD unit (Rockville, Maryland, United States). Extra precautions were taken to ensure the samples never dried during the washing, dehydration and CPD process. The samples were mounted on imaging pedestals and carbon coated using Cressington 208carbon High-Vacuum Carbon Coater (Watford, England, Unite Kingdom). HIM imaging was performed using a Zeiss Orion Plus Helium Ion Microscope (Oberkochen, Germany) at the Pacific Northwest National Laboratory (PNNL) Environmental Molecular Sciences Laboratory (EMSL).

### Detection of surface functional group changes: ATR-FTIR

Marine exposed plastic samples and terrestrial UV exposed controls were collected from each time point to observe the formation of chemical function groups on the surface of the polyolefins. To control for natural UV irradiation and carbonyl development, UV controls for each polymer type were deployed on the BIOS facility roof and retrieved in parallel with the collection of the marine samples. Unexposed controls were kept at ambient temperature in the packaging they were received in throughout the experiment and processed in the same manner as the marine and nature UV samples at each timepoint. Post incubation, attached biofilms were digested in 0.2 μm filtered 10% KOH for 48 h, shaken at 80 rpm and rinsed with 18.2 MΩ·cm filtered DI water to reduce signals related to organic matter accumulation. The samples were analyzed on a Thermo-Fisher Nicolet iS20 FTIR (Waltham, MA, United States). Using a diamond tip, each sample was scanned 64 times to measure Attenuated Total Reflectance (ATR) and Bruker OPUS software was used to plot the spectral data. Atmospheric corrections were collected, applied to every sample spectrum to reduce background noise. The spectra were processed using Open Specy with default smoothing (polynomial setting 3) and baseline correction (polynomial setting 8) applied, a plastics-specific spectral database, to reassess and verify sample identity (Cowger et al., 2021). To assess whether the marine exposed polymers sustained significant surface changes, differences between the mean treatment (marine incubated, natural UV, unexposed) values associated with each polymer type were conducted using one-way ANOVA tests (alpha = 0.05), homogeneity of variance assessed (Levene’s test) and between-group statistical significance identified using post-hoc tests (Tukey or Welsh F test). Carbonyl index is the ratio of ATR-FTIR measured absorbance of the area under band (AUB) of carbonyl peaks range (1,850–1,650 cm^−1^), divided by the AUB of the CH2 peaks (1,500–1,420 cm^−1^) as described by Almond et al. (2020). Terminal C=C bond ratios for each polymer type and treatment were generated using method described by Hadar and Sivan (2004) and Tribedi and Dey (2017). PE and PP percent crystallinity values were calculated using equations described by Tarani et al. (2023) and Lanyi et al. (2018), respectively.

### DNA processing, sequencing, and assignment of ASVs

Bacterial DNA extraction of material dislodged cells and free-living seawater were performed using the DNeasy Blood and Tissue DNA extraction kit (Qiagen; Hilden, Germany). Extracted DNA products were stored at −20°C until further processing was performed. Amplification of the 16S rDNA V4 hypervariable region was performed using the 515F – 806R primers and Platinum Hot Start PCR Mix (ThermoFisher; Waltham, Massachusetts) following the Earth Microbiome Project 16S rDNA protocol (Caporaso et al., 2018). PCR products purification was performed using the QIAquik purification kit (Qiagen; Hilden, Germany). Normalized purified libraries were processed on an Agilent High Sensitivity D5000 ScreenTape System (Agilent Technologies; Santa Clara, California, United States) by Center for Quantitative Life Sciences (CQLS) at Oregon State University to ensure the amplified products were representative of the target sequence. Sequencing was subsequently performed by the CQLS on the 2 × 300 bp Illumina MiSeq V3 platform (Illumina; San Diego, California, United States).

Sequence processing and taxonomic classification was performed using dada2 (v. 1.8) which implements the Ribosomal Database Project (RDP) Naive Bayesian Classifier (NBC) algorithm described in Wang et al. (2007) to assign taxonomy across Linnean taxonomic levels (i.e., Kingdom to Genus) using the Silva taxonomy training set (nr99, v. 138.1) (Callahan et al., 2016; McLaren and Callahan, 2021). Amplicon sequence variant (ASV) tables were generated following the Davis et al. (2022) dada2 documentation. The Decipher package (v. 2.26.0) was also used to generate putative taxonomic classifications (Wright, 2016). To ensure optimal taxonomic classification, both pipelines were applied using the Silva and GTDB training sets and the resulting taxonomic assignments were compared based upon the number of unassigned taxa (Quast et al., 2012; Parks et al., 2022). The 16S rDNA V4 sequences used in each comparison were trimmed with acceptable sequence quality score (≥ 20), read depth (≥ 30,000) and chimeras removed. The dada2 pipeline and Silva training set was used as it resulted in the least number of NAs present at the Order and Genus level (1885, 4,804), followed by Decipher + Silva (3,079, 6,051) and Decipher + GTDB (3,100, 4,823). The phyloseq package (v. 1.42.0) was used to analyze, compile, and graphically interact with the phylogenetic sequencing data curated by the dada2 package and associated sample data (McMurdie and Holmes, 2013).

### Phylogenetic refinement via cladal taxonomic annotation

The ASVs which failed to be taxonomic annotated were subjected to a novel taxonomic annotation approach called Cladal Taxonomic Annotation (CTA), which expands on our previous work (Gaulke et al., 2018; Sharpton et al., 2021; Arnold et al., 2022). We used the All Species Living Tree Project (LTP) type strain 16S sequences and taxonomy (v. LTP_06_2022) as reference for our phylogenetic tree generation (Pruesse et al., 2012). Briefly, we used the SINA aligner (v. 1.7.2; settings: -t --search --lca-fields = tax_ltp, tax_gtdb, tax_embl_ebi_ena--fasta-write-dna --preserve-order -p 32) to align our ASVs and LTP reference sequences. Columns with 100% gap sequences were removed using trimal (v 1.4) (setting: -noallgaps) (Capella-Gutiérrez et al., 2009). The aligned sequences were used as input to FastTreeMP 2.1.11 (settings: OMP_NUM_THREADS = 32 -nt -gtr) with the generalized time reversible (GTR) model of nucleotide substitution (Price et al., 2010). The resultant phylogenetic tree of ASVs and reference sequences was midpoint rooted (phangorn v. 2.11.1; midpoint function; Schliep, 2011). Prior to performing CTA, we resolved several homotypic synonyms using the NCBI Taxonomy Browser (Schoch et al., 2020), so that Linnean taxonomic labels assigned at a particular taxonomic level would be identical despite taxonomic label incongruencies from historical discrepancies (e.g., Phylum level “Planctomycetota” and “Planctomycetes”). ASVs which failed taxonomic assignment with traditional methods at higher taxonomic levels (e.g., Phylum, Class) were assigned using the CTA algorithm. The CTA algorithm works by propagation of known taxonomic labels of reference tips to unknown ASVs by assigning each internal node within the phylogenetic tree with the most specific taxonomic labels that results in taxonomic congruence of all known descendent tips (Supplementary Figure S6).

After tree generation, ClaaTU (v. 0.1) was used to assign taxonomic labels to each of the nodes to the tree based on a modified majority rule guideline (Gaulke et al., 2018). Only the LTP reference sequence taxonomic classifications were used to assign labels to the nodes. To assign taxonomic labels to the ASVs, nodes were traversed from each ASV tip until a node with taxonomic assignment is found. The reporting of ClaaTU-assigned ASV taxonomic classifications in this study were only performed in instances where the dada2 classifier failed. The taxonomic source of annotations (e.g., NBC vs. CTA) is noted throughout the manuscript to convey our certainty of taxonomic assignment (i.e., more certainty lies with those taxonomic annotations prescribed by the traditional NBC vs. our CTA).

### Statistical analysis of community composition, differential abundance, and figure generation

**Figure 5:**
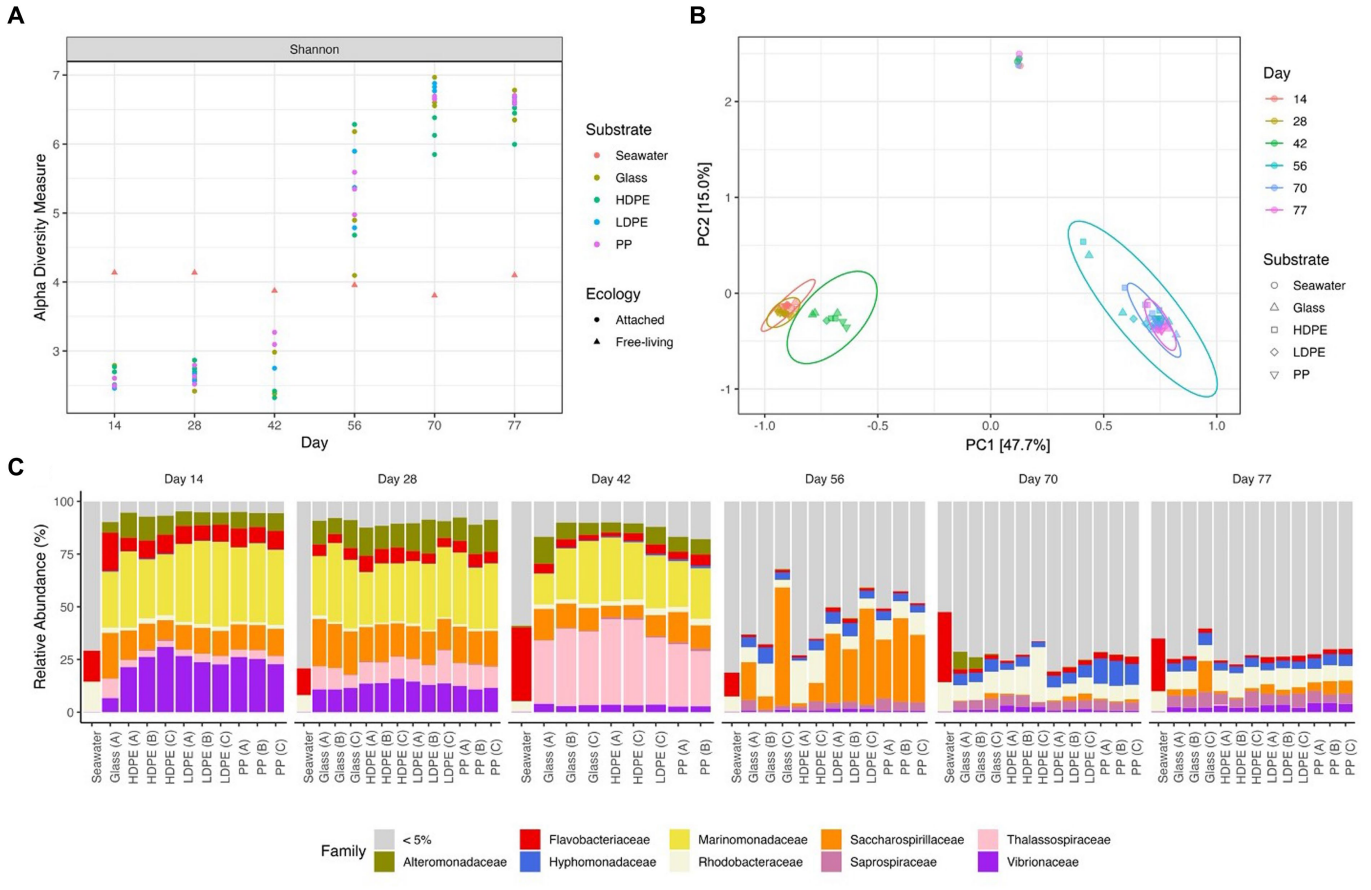
Gradual temporal shift in a/b diversity shared among material colonizing communities. The Shannon alpha diversity plot **(A)**, Bray-Curtis PCoA (MDS) ordination **(B)**, and Relative abundance stacked bar chart **(C)** showcase the transition in community complexity and inter-, intra-group similarity over time. In plot **(A)**, alpha diversity measures of the substrate attached communities sharply increases following the mid-experimental transition (between days 42 and 56). Plot **(B)** explores the compositional dissimilarity of the microbial communities (9,069 unique ASVs) present on the plastics, glass and seawater over the incubation period based on a Bray-Curtis distance matrix. Plot **(C)** shows the community composition of the top 5% taxa present in each substrate type throughout the incubation period.

Using the adonis2 (permanova), betadisper and ANOSIM functions within the vegan package (v. 2.6–4), permutational tests for homogeneity of multivariate variance and dispersions were conducted (Oksanen et al., 2013). MicroViz (v. 0.10.0) and phyloseq packages were used for data curation and figure generation pertaining to a/b diversity (McMurdie and Holmes, 2013; Barnett et al., 2021). The Shannon diversity index was used to measure community alpha diversity measures (Figure 5A), and the degree of dissimilarity in beta diversity of the various bacterial communities was measured using a Principal Component Analysis (PCoA) ordination of compositional data with unique taxonomic rank based on a Bray-Curtis distance matrix (Figure 5B). The DESeq2 package (v. 1.38.2) was used to produce differential abundance assessments across substrate types and time. Each of the resulting assessments were validated using the Wald Test with Benjamini-Hochberg adjustment (adj. *p* ≤ 0.05) to produce *p*-values for each ASV (Love et al., 2014). To identify the most confident differential abundances, the comparisons were performed using data from time points that possessed at least three biological replicates. Differential abundance assessments and log2foldchange plots of colonizing taxa were performed across substrate types and time, and validated using Wald Test with Benjamini-Hochberg adjusted *p* values (adj. *p* ≤ 0.05) for each ASV. Using Log2FoldChange differential abundance data of significantly enriched taxa associated with polyolefins in comparison to glass (DESeq2) and taxonomic information for each ASV, the heat tree matrix was created using the Metacoder (v. 0.3.6) program (Foster et al., 2016). Following taxonomic assignment of ASVs using CTA, reference sequences were dropped from the tree and were visualized using ggtree (v. 3.6.2; Yu et al., 2017). To determine the most ancestral root that shared a particular taxonomic classification label, a custom script was used to remove all nested nodes, resulting in the most ancestral node that is shared by all members labeled to a particular taxonomic level (R v. 4.2.1) and was layered with annotations using ggtreeExtra (v. 1.10.0) and ggnewscale (v. 0.4.9; Yu et al., 2017).

### Functional analysis of polyolefin colonizing taxa

Functional analysis of bacterial community member functions was performed using the FAPROTAX database in association with the Microeco program, using 16S rDNA (V4) sequences as input data, with standard settings (Louca et al., 2016; Liu et al., 2021). The functional annotations from this analysis were obtained by mapping 16S rDNA genus-level identifications to functions described in the literature from experiments with cultured representatives.

### Data availability

The raw 16S (V4) rDNA sequences generated can be accessed through the NCBI Sequence Read Archive (accession: PRJNA1005706). The code used in this report for sequence curation and figure generation is available at: https://github.com/singleseOSU/2021-Bermuda-Polyolefin-16S-Investigation.

## Results

### Statistical analysis of community composition influential factors

To determine the significance of experimental variables on community assemblage, permanova, beta dispersion and ANOSIM analyses were performed (Table 1). Unexpectedly, we observed a prominent significant transition in the most abundant biofilm taxa between days 42 and 56; therefore, we refer to an ‘early’ period (days 14–42) and a ‘late’ period (days 56–77) to describe the community composition. Period and incubation time were found to have the largest significant influence on the microbial communities throughout the exposure, whereas material type did not significantly influence microbial community composition. However, when material type was observed as an interaction term paired with incubation time, material type accounted for 13% of the variation in community composition at specific incubation times. When exploring the material-type time-dependent significance, it was found that polymer type had a significant effect in the late period, but not the early period. This may be due to the abrupt increase in species richness of the community with the rise of rare (≤1% relative abundance) colonizing taxonomic groups in the late period.

**Table 1:** Statistical analysis of influential factors on substrate-attached community composition.

Test	Variable	Significance
Permanova	Incubation time	*p* = 0.001; *R*^2^ = 0.684; *F* = 34.25 ***
	Incubation time: period (early)	*p* = 0.001; *R*^2^ = 0.609; *F* = 21.02 ***
	Incubation time: period (late)	*p* = 0.001; *R*^2^ = 0.236; *F* = 4.936 ***
	Incubation time: material type	*p* = 0.001; *R*^2^ = 0.133; *F* = 2.579 ***
	Period	*p* = 0.001; *R*^2^ = 0.570; *F* = 62.28 ***
	Material type	*p* = 0.788; *R*^2^ = 0.055; *F* = 0.219
	Material type: period (early)	*p* = 0.096; *R*^2^ = 0.057; *F* = 1.88
	Material type: period (late)	*p* = 0.001; *R*^2^ = 0.178; *F* = 15.66 ***
	Ecology (pelagic vs. attached)	*p* = 0.001; *R*^2^ = 0.160; *F* = 13.30 ***
Permutest (Betadispersion)	Incubation time	*p* = 0.001; *F* = 13.35 ***
	Incubation time: period (early)	*p* = 0.806; *F* = 0.232
	Incubation time: Period (late)	*p* = 0.704; *F* = 0.401
	Incubation time: material type	*p* = 0.001: *F* = 4.032 ***
	Period	*p* = 0.001; *F* = 58.96 ***
	Material type	*p* = 0.804; *F* = 0.339
	Material type: period (early)	*p* = 0.857; *F* = 0.264
	Material type: period (late)	*p* = 0.398; *F* = 1.032
	Ecology (pelagic vs. attached)	*p* = 0.001; *F* = 106.1 ***
ANOSIM	Incubation time	*p* = 0.001; *R* = 0.586 ***
	Period	*p* = 0.001; *R* = 0.676 ***
	Material type (late)	*p* = 0.001; *R* = 0.552 ***
	Material type (day 56)	*p* = 0.003; *R* = 0.542 ***
	Material type (day 70)	*p* = 0.001; *R* = 0.756 ***
	Material type (day 77)	*p* = 0.001; *R* = 0.661 ***
	Polymer type (day 56)	*p* = 0.004; *R* = 0.796 ***
	Polymer type (day 70)	*p* = 0.004; *R* = 0.835 ***
	Polymer type (day 77)	*p* = 0.004; *R* = 0.794 ***
	Ecology (pelagic vs. attached)	*p* = 0.001; *R* = 0.222 ***

Consistent with this supposition, the relative abundance of rare taxa increased overtime, from 28.9% in the early period to 83.7% in late period samples. When tested for beta dispersion, incubation time, period, and material type, all did not differ in beta-dispersion as a function of incubation time. An ANOSIM analysis found supporting evidence of significant dissimilarities between the seawater and material-bound communities, incubation time and significant dissimilarity between material types in the late period. As previously mentioned, there were no significant difference observed between the polymer types or glass during the early period, but as time progressed the community diversity sharply increased and substrate type gained weak significance in the late period. Although the metrics used account for rare taxa (but not highly weighted) within the communities, this observation led us to look past the abundant colonizers and focus on the rare taxa associated with each plastic type by assessing the differential abundance compared between glass and plastic type. As we presumed that the increase collective relative abundance of rare taxa within these biofilm communities were driving the between-group dissimilarity.

### Plastic community composition and diversity varies considerably over time

Early period communities were characterized by members of the Gammaproteobacteria Alphaproteobacteria, and Bacteroidia classes. Their abundance continued throughout the exposure into the late period, at which they were joined by the emergence of abundant taxa in the Verrucomicrobiae, Planctomycetes, Bdellovibrionia, Polyangia and Oligoflexia. Note the reported relative abundances in this passage are listed based on change over time and further reported in Table 2. By day 77, the polymer attached communities significantly increased in phylogenetic diversity. Throughout the exposure, the seawater community was steadily dominated by members of the Alphaproteobacteria, Bacteroidia, Gammaproteobacteria and Cyanobacteria. At the family-level, Marinomonadaceae, Vibrionacaeae, Thalassospiraceae, Saccharospirillaceae, Alteromonadaceae and Flavobacteriaceae were the most abundant plastic colonizing lineages. At the genus-level within these families, *Marinomonas*, *Vibrio*, *Thalassospira*, *Oleibacter*, and *Alteromonas* were the most abundant. Successional changes in the communities were slow until an abrupt transition occurred at day 42. By day 56, most of the early abundant taxa had declined below the 5% abundance threshold and some to nearly undetectable levels (Figure 5). The late period can be characterized as a period of rapid diversification, where the number of highly abundant lineages decline, and a myriad of ‘rare’ taxa rise. Following the transition, Saccharospirillaceae, Rhodobacteraceae, Hyphomonadaceae, Saprospiraceae, Vibrionaceae, and Flavobacteriaceae were the few families with >5% relative abundance. Whereas at the Genus-level, *Saccharospirillum, Vibrio* and *Synechococcus* were the few remaining genera with >1% relative abundance. Most of the colonizing genera were rare community members (≤1% relative abundance).

**Table 2:** Abundant polyolefin colonizing lineages across time periods.

Class	ASVs (#)	Period	Average relative abundance (%)
Gammaproteobacteria	366/1,161	Early/Late	71.5/32.7
Alphaproteobacteria	623/1,762	Early/Late	18.7/32.5
Bacteroidia	313/793	Early/Late	8.5/12.3
Verrucomicrobiae	34/151	Early/Late	0.06/3.6
Cyanobacteria	23/67	Early/Late	0.09/3.2
Planctomycetes	18/91	Early/Late	0.01/2.9
Bdellovibrionia	26/79	Early/Late	0.12/2.3
Polyangia	42/126	Early/Late	0.07/2.3
Oligoflexia	25/196	Early/Late	0.04/1.7
**Family**	**ASVs (#)**	**Period**	**Average relative abundance (%)**
Alteromonadaceae	10	Early	8.9
Marinomonadaceae	3	Early	30.4
Thalassospiraceae	1	Early	18.5
Flavobacteraceae	16	Early/Late	0.8/1.1
Saccharospirillaceae	7	Early/Late	13.3/2.1
Vibrionaceae	8/6	Early/Late	14.1/1.8
Hyphomonadaceae	38	Late	3.9
Saprospiraceae	42	Late	2.4
Cyanobiaceae	12	Late	1.3
Rhodobacteraceae	108	Late	5.7
Oligoflexaceae	131	Late	1.5
**Genus**	**ASVs (#)**	**Period**	**Average relative abundance (%)**
*Marinomonas*	3	Early	30.4
*Thalassospira*	1	Early	18.5
*Oleibacter*	1	Early	10.0
*Alteromonas*	1	Early	7.7
*Vibrio*	8/6	Early/Late	14.1/1.8
*Saccharospirillum*	4	Late	9.9
*Synechococcus*	7	Late	1.1
*Hyphomonas*	4	Late	0.8

### Material-associated microbiomes differ from ambient seawater communities

As expected, seawater microbial communities were dominated by well-known planktonic taxa and were distinctly different from attached communities (Figure 5). We assumed the seawater would harbor a bacterial community dissimilar to the material surfaces and that differences would be seen between the glass and polyolefins. To explore the predictions that polymer type would influence community composition, and that early colonizing taxa would be gradually outcompeted by subsequent colonizers over the duration of the experiment, alpha and beta diversity were measured and compared. As expected, the polyolefin-attached communities were more similar to the glass than the seawater communities and more influenced by time than by substrate (*p*_Time_ < 0.05 < *p*_Substrate_). The observation of community succession on the polyolefins is in accord with other reports (Tu et al., 2020; Wright et al., 2020; Cheng et al., 2021). The influence of polymer type on community structure was only slightly significant in the late period, supported by permanova (*p* < 0.05) and beta dispersion (*p* > 0.05) tests. The small differences observed between polymer types was the product of rare taxa presence/absence as community alpha diversity increased over time (Figure 5A).

A ‘mid-experimental’ community transition was observed on the polyolefin and glass samples but not in the surrounding seawater community (Figure 5). Community assemblages changed between days 42 and 56, which resulted in dramatic increases in community diversity of the biofilms, as seen in the ɑ-diversity plots and compositional Bray-Curtis PCoA ordination. Bacterial richness on the surfaces increased at this time (Figure 5A). Before the transition, richness was lower in the biofilms compared to the ambient seawater, which aligns with the reports of mesoplastic-colonizers of unknown marine residence time by Debroas et al. (2017). Following the transition, bacterial community richness abruptly increased resulting in values higher than the ambient seawater in accord with reports by Bryant et al. (2016) and De Tender et al. (2015).

### Polyolefins uniquely recruit specific microbial taxa

The ability of marine bacteria to degrade highly recalcitrant forms of carbon such as polyolefin plastics was once considered unlikely, in part because these materials have existed on Earth for too short a time to support the evolution of specialized enzymatic machinery for their degradation. However, in recent decades there has been a rise in reports of bacterial degradation of multiple plastic types (Hadar and Sivan, 2004; Sudhakar et al., 2008; Balasubramanian et al., 2010; Harshvardhan and Jha, 2013; Santo et al., 2013; Yoshida et al., 2016; Paço et al., 2017; Syranidou et al., 2017; Auta et al., 2018; Mohanrasu et al., 2018; Devi et al., 2019; Oberbeckmann and Labrenz, 2019; Roager and Sonnenschein, 2019; Dey et al., 2020; Elumalai et al., 2021; Gao and Sun, 2021). It has been hypothesized that the degraders of natural hydrocarbons may play a role in plastic degradation in the marine environment (Roager and Sonnenschein, 2019). Although they were not significantly associated with one polyolefin over another in our study, a few of the most abundant colonizing taxa from various time points, *Marinomonas*, *Oleibacter, Alteromonas, Thalassospira and Hyphomonadaceae*, harbor members that have been described to possess aromatic hydrocarbon and/or alkane degradation capabilities (Teramoto et al., 2011). Many of these bacteria are ubiquitous in marine environments as generalist or obligate hydrocarbonoclastic organisms (e.g., *Thalassospira* and *Oleibacter*; Head et al., 2006; Yakimov et al., 2007, 2019, 2022).

**Figure 6:**
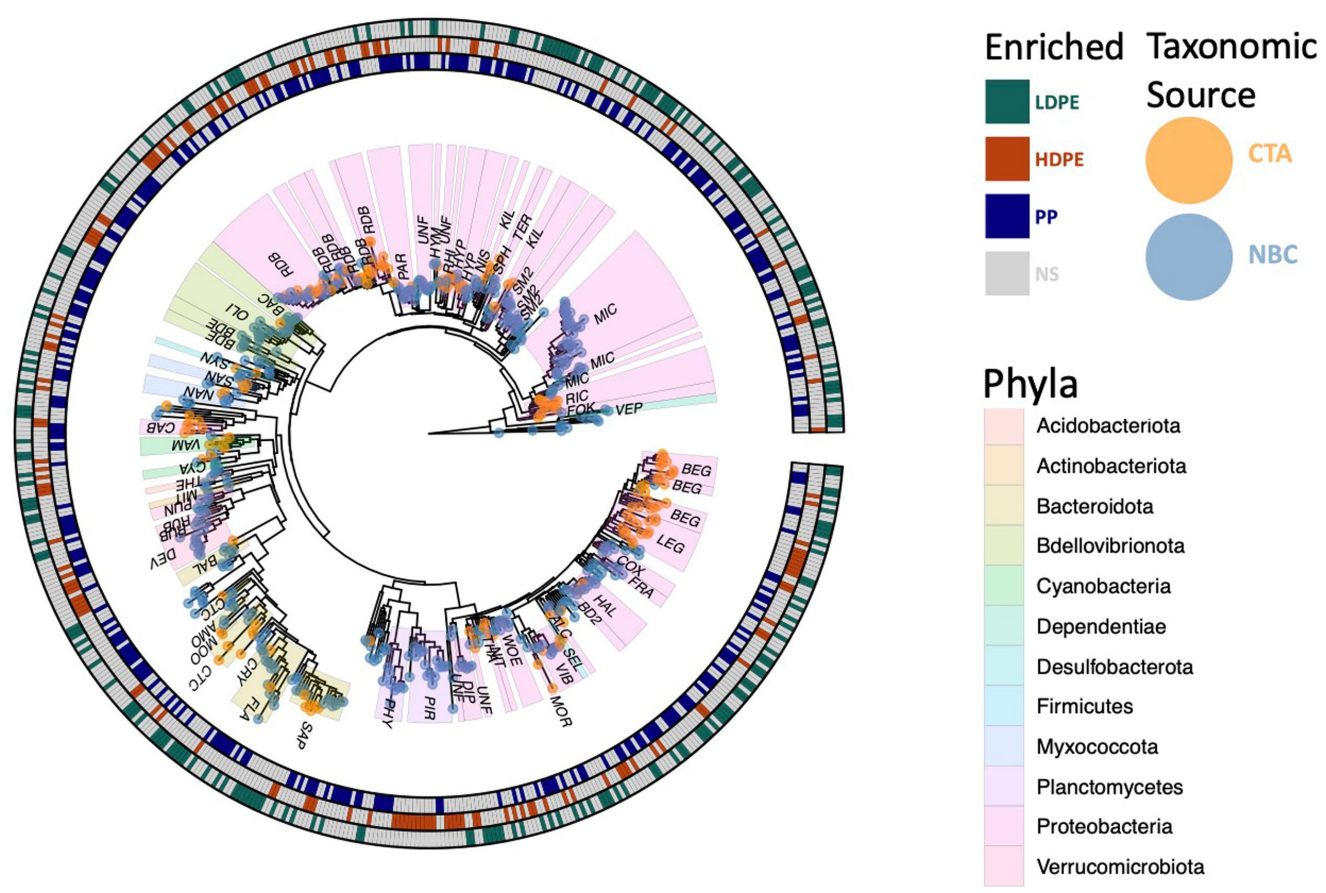
Phylogenetic tree of polyolefin enriched ASVs. All 594 significantly enriched taxa observed on one or more polymer types in comparison to the glass control at various time points throughout the incubation are shown. Outer circle colors indicate taxa polymer specificity, and the tip colors denote taxonomic classification source used (NBC, naïve bayesian classifier; CTA, cladal taxonomic annotation). Three digit codes identify family lineages: Alcanivoracaceae (ALC), Amoebophilaceae (AMO), BD2-7 (BD2), Bacteriovoracaceae (BAC), Balneolaceae (BAL), Beggiatoaceae (BEG), Bdellovibrionaceae (BDE), Caedibacteraceae (CAB), Coxiellaceae (COX), Cryomorphaceae (CRY), Cyanobiaceae (CYA), Cyclobacteriaceae (CTC), Diplorickettsiaceae (DIP), DEV007 (DEV), Flavobacteriaceae (FLA), Fokiniaceae (FOK), Francisellaceae (FRA), Halieaceae (HAL), Hyphomonadaceae (HYP), Hyphomicrobiaceae (HYM), Kiloniellaceae (KIL), Legionellaceae (LEG), Micavibrionaceae (MIC), Microtrichaceae (MIT), Mooreiaceae (MOO), Morganellaceae (MOR), Nannocystaceae (NAN), Nisaeaceae (NIS), Nitrosococcaceae (NIT), Oligoflexaceae (OLI), Parvularculaceae (PAR), Phycisphaeraceae (PHY), Pirellulaceae (PIR), Rhizobiaceae (RHI), Rhodobacteraceae (RDB), Rickettsiaceae (RIC), Rubritaleaceae (RUB), SM2D12 (SM2), Sandaracinaceae (SAN), Saprospiraceae (SAP), Selenomonadaceae (SEL), Sphingomonadaceae (SPH), Syntrophorhabdaceae (SYN), Terasakiellaceae (TER), Thermoanaerobaculaceae (THE), Thioalkalispiraceae (THA), Unknown Family (UNF), Vampirovibrionaceae (VAM), Vermiphilaceae (VEP), Vibrionaceae (VIB), Woeseiaceae (WOE).

**Figure 7:**
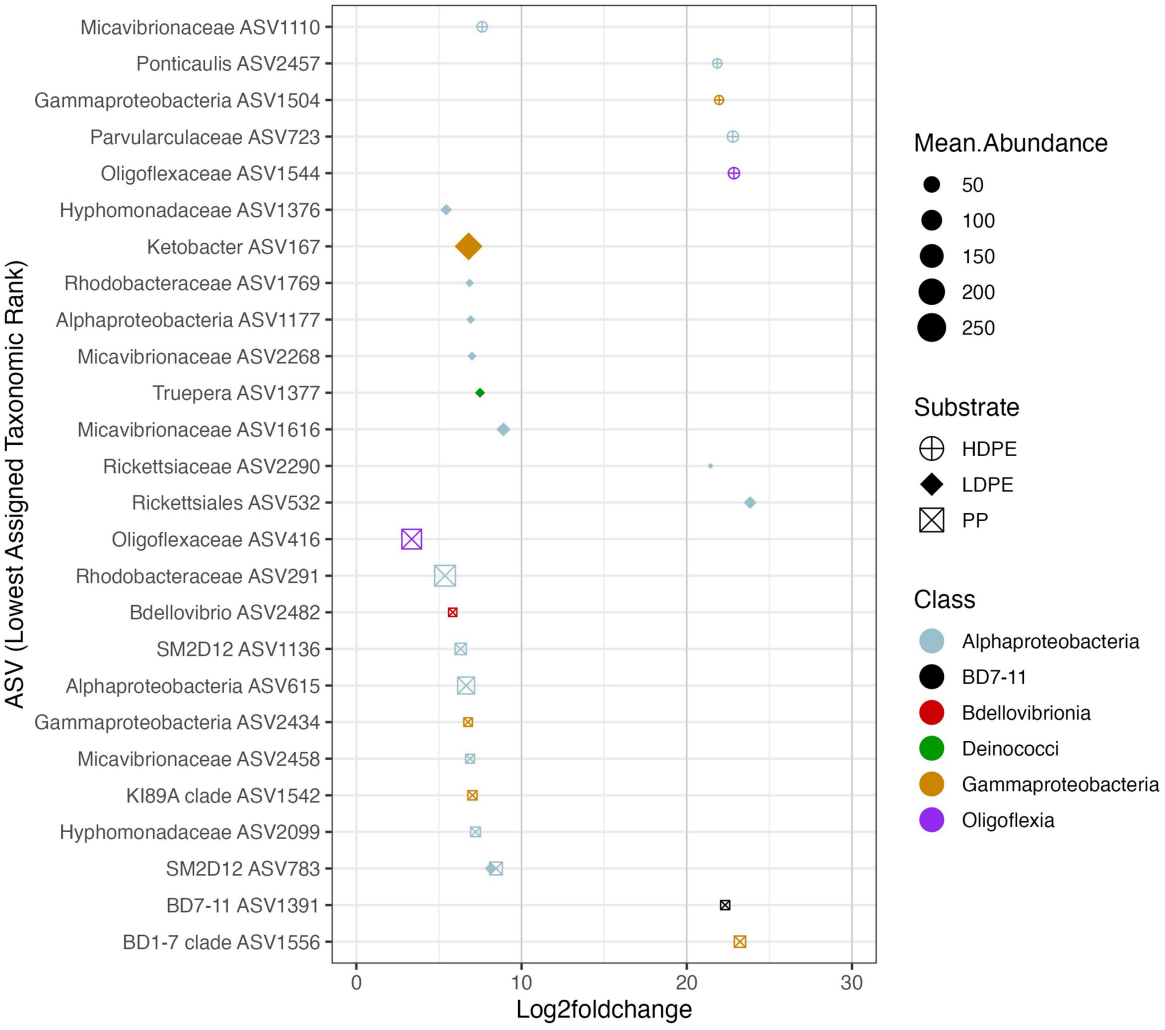
Polymer enriched marine taxa. The Log2foldchange plot showcases NBC classified ASVs that were significantly enriched (*p*_adj_ ≤ 0.05) on either one or more polymer types throughout the incubation. The color, size and shape of the data points are associated with the enriched taxon’s class, mean abundance, and substrate preference, respectively. Mean abundance is the average of the sequence depth normalized count values for all included samples, whereas Log2FoldChange is the effect size estimate. All ASVs listed possess >3 log fold differences in abundance compared to glass. Day 42 (and 56 for HDPE) Log2FoldChange data were not included due to loss of sample replicates at the time point, similar rationale was used for Day 14 for all three polymers in respect to the loss of glass control biological replicates.

To determine which of the 8,641 polymer-colonizing taxa were significantly enriched by substrate composition over the entire exposure period, differential abundance assays were conducted using DESeq2 (Barnett et al., 2021). Five hundred and ninety-four significantly enriched ASVs were observed on the polymers at various time points (Figure 6). 26 colonizing ASVs were present throughout the entire study period and of those 25 were observed to be enriched on specific polymer types (Figure 7). When observing the material-specific significant enriched ASVs, the amount that were significantly enriched on glass was consistently greater compared those significant enriched on each of the various plastics (glass vs. HDPE [48|5], glass vs. LDPE [19|10], glass vs. PP [32|12]). Inert glass is thought to offer no comparative nutritional benefits to colonizing taxa other than providing a charged surface for attachment (Dudek et al., 2020). Whereas plastic polymers potentially offer colonizing cells organic carbon in recalcitrant forms, and additional beneficial or hazardous compounds depending on the degradation state of the polymer, production additives (e.g., phenolic/phosphite antioxidants, stearate stabilizers, protective coatings) and chemicals absorbed from the environment (e.g., PAHs, BPA, DDT; Hirai et al., 2011; Hahladakis et al., 2018; Liu et al., 2019). Depending on these factors, marine incubated plastics may select against colonization by various lineages of rare marine bacterial taxa.

### Reclassification of unclassified polymer enriched ASVs via taxonomic agglomeration

A significant proportion the DESeq2 identified 594 significantly enriched ASVs could not be accurately classified due to the relatively large distance from closest neighbors in the Silva dataset (v. 138.1; McLaren and Callahan, 2021). We hypothesized that the lack of taxonomic annotations was due to the closest neighbors to the ASVs in the Silva database having low-quality or no taxonomic annotations themselves. We confirmed this hypothesis by using the unclassified ASVs as query sequences in the NCBI database web interface (Schoch et al., 2020). The vast majority of the ASVs returned neighbors that were of environmental and/or metagenomic origin and did not have taxonomy assignments. To account for this, we opted to use a method of taxonomic agglomeration called Cladal Taxonomic Annotations (CTA), which (Zalasiewicz et al., 2017) applies known taxonomic annotations of reference tips to propagate information about taxonomy to ancestral internal nodes within the phylogenetic tree and then (Barnes et al., 2009) subsequently assigns taxonomic labels to unknown ASVs by using the taxonomic label of the most recent common ancestor (Supplementary Figure S7; Gaulke et al., 2018; McLaren and Callahan, 2021; Arnold et al., 2022). Given the novelty of the CTA method we first labeled all figures with taxonomic annotations obtained using RDP NBC if available, and then only subsequently annotated unknown nodes with CTA. Following the reclassification, we found that there were large parts of enriched community biodiversity which were associated with lineages that lacked adequate taxonomic representatives within the NBC database, most notably within the Ehrlichiaceae and Legionellaceae families. Of the 594 NBC classified ASVs, 171 (29%) were reclassified with CTA annotations. The CTA method produced greater taxonomic insight into multiple poorly classified ASVs of interest, which include 8 of the 25 polyolefin-specific significant enriched ASVs (Table 3).

**Table 3:** List of NBC classified, and CTA reclassified taxonomic assignments of polyolefin-specific significantly enriched ASVs.

Tax source	ASV	Class (NBC)	Family (NBC)	Genus (NBC)	Family reclassified	Genus reclassified
CTA	291	Alphaproteobacteria	Rhodobacteraceae	NA	Rhodobacteraceae	*Albimonas*
CTA	532	Alphaproteobacteria	NA	NA	Fokiniaceae	*Lyticum*
CTA	615	Alphaproteobacteria	NA	NA	Rhodobacteraceae	*Albimonas*
CTA	1376	Alphaproteobacteria	Hyphomonadaceae	NA	Hyphomonadaceae	*Ponticaulis*
CTA	1504	Gammaproteobacteria	NA	NA	Nitrosococcaceae	*Inmirania*
CTA	2290	Alphaproteobacteria	Rickettsiaceae	NA	Rickettsiaceae	*Rickettsia*
CTA	2434	Gammaproteobacteria	NA	NA	Beggiatoaceae	*Thiomargarita*
NBC	167	Gammaproteobacteria	Alcanivoracaceae	*Ketobacter*	No change	No change
NBC	416	Oligoflexia	Oligoflexaceae	NA	No change	No change
NBC	723	Alphaproteobacteria	Parvularculaceae	NA	No change	No change
NBC	1110	Alphaproteobacteria	Micavibrionaceae	NA	No change	No change
NBC	1136	Alphaproteobacteria	SM2D12	NA	No change	No change
NBC	1177	Alphaproteobacteria	NA	NA	No change	No change
NBC	1391	BD7-11	NA	NA	No change	No change
NBC	1377	Deinococci	Trueperaceae	*Truepera*	No change	No change
NBC	1542	Gammaproteobacteria	KI89A clade	NA	No change	No change
NBC	1544	Oligoflexia	Oligoflexaceae	NA	No change	No change
NBC	1556	Gammaproteobacteria	Spongiibacteraceae	BD1-7 clade	No change	No change
NBC	1616	Alphaproteobacteria	Micavibrionaceae	NA	No change	No change
NBC	1769	Alphaproteobacteria	Rhodobacteraceae	NA	No change	No change
NBC	2099	Alphaproteobacteria	Hyphomonadaceae	NA	No change	No change
NBC	2268	Alphaproteobacteria	Micavibrionaceae	NA	No change	No change
NBC	2457	Alphaproteobacteria	Hyphomonadaceae	*Ponticaulis*	No change	No change
NBC	2458	Alphaproteobacteria	Micavibrionaceae	NA	No change	No change
NBC	2482	Bdellovibrionia	Bdellovibrionaceae	*Bdellovibrio*	No change	No change

### Plastic types differ in the ‘rare’ taxa they recruit

**Figure 8:**
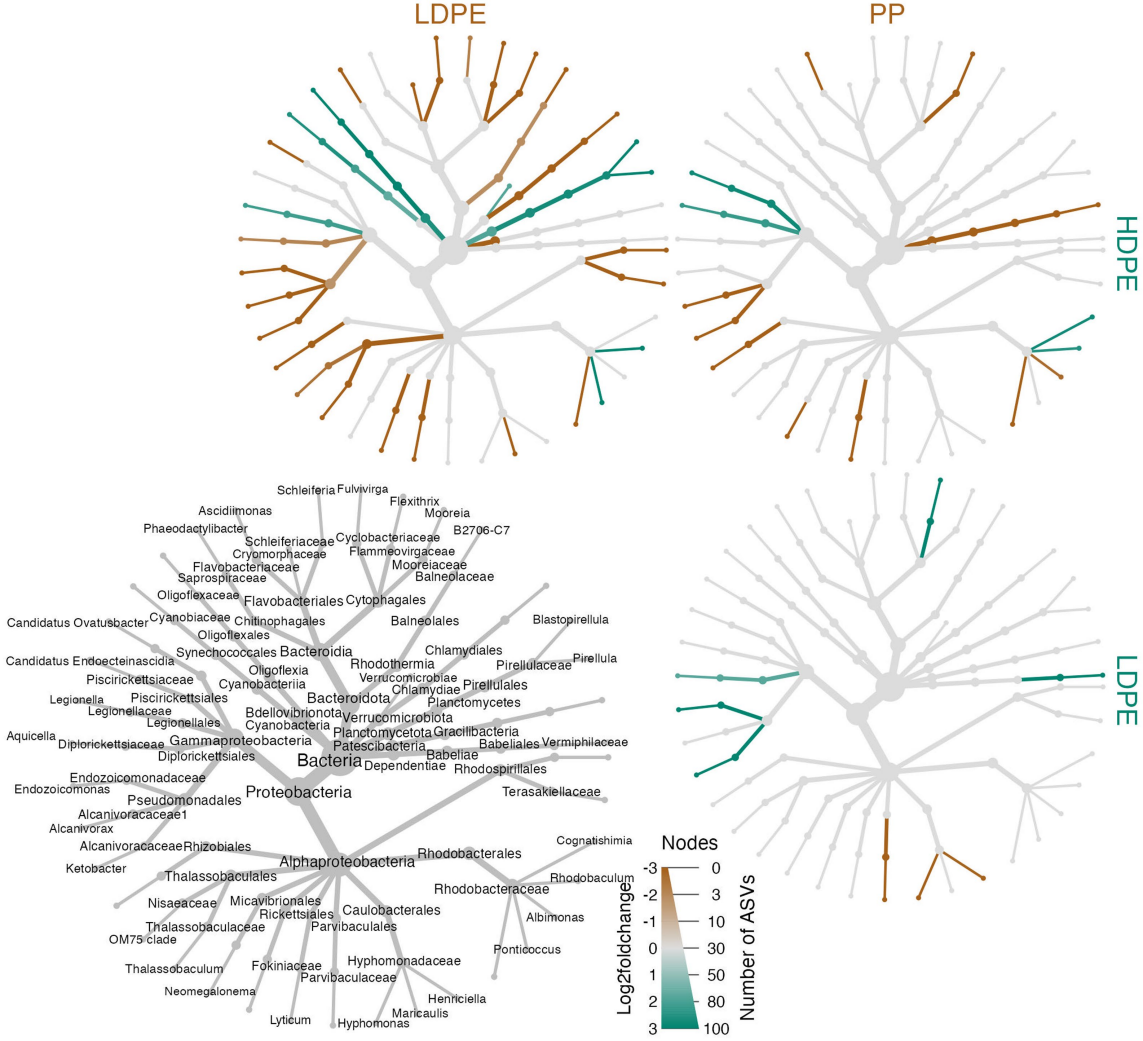
Heat tree matrix of significant polymer-enriched taxa. The strength of an enrichment (Log2FoldChange) is indicated by the color gradient assigned to the node/edge (brown, gray, green; gray = no significant difference) and node size reflects the number of ASVs within the group. Further, the compared materials and associated enriched taxa are indicated by the color of the material label. The heat trees showcase a two-way Log2FoldChange comparisons of the taxa enriched on the different polyolefins over the incubation period. A legend tree located in the bottom left corner denotes taxonomy for all trees shown to the lowest classified rank (nodes missing labels are indicative of poor taxonomic resolution at that rank).

To ask whether taxa enriched on polyolefins relative to glass were specific to a particular polyolefin, the differential abundance analysis was repeated using a dataset that contained only polyolefin abundance data for the taxa that were significantly enriched on the plastics in comparison to the seawater and glass. As detailed previously, in community-wide comparisons of taxon abundance by material type (including glass) there were no significant differences in the overall community because the most abundant taxa were shared between communities. However, when observing the representation of rare taxa (≤1%), few taxa were found to be significantly associated with one polymer type over another (Figures 6–8). When comparing the sets of significantly enriched taxa that resulted from each polymer-glass comparison, most taxa were shared between the polymer types, but 25 ASVs were found to be significantly associated with one polymer over the others throughout the incubation (Table 2). Five were specifically enriched on HDPE (ASV723, ASV1110, ASV1504, ASV1544, ASV2457), 9 on LDPE (ASV167, ASV532, ASV1177, ASV1376, ASV1377, ASV1769, ASV2268, ASV1616, ASV2290), and 11 on PP (ASV291, ASV416, ASV615, ASV1136, ASV1391, ASV1542, ASV1556, ASV2099, ASV2434, ASV2458, ASV2482) (Table 3; Figure 7). Lineages that were significantly enriched on multiple polymers included 13 ASVs within the Oligoflexaceae (HDPE, PP), 15 Hyphomonadaceae (all polymers) and 58 Rhodobacteraceae (all polyolefins) families.

### Diverse microbial phenotypes observed on polyolefin surfaces

To characterize the microbe-polyolefin interface and identify artifacts of microbial biodegradation artifacts (formation of irregular pits and/or cracks) following the incubation, images of the polyolefins were captured using HIM. A variety of ecosystem members and biofilm characteristics were documented on a visual range of 100 μm to 10 nm on all polymers (Figures 2–4). Saprotrophs (fungal hyphae), phototrophs (pennate diatoms), heterotrophs (various coccoid and bacilli) and dense network of extra polymeric substance (EPS) webbing and projections were nearly ubiquitous. Known to be produced by phase 2 secondary colonizers, cellular projections in the form of EPS of varying lengths (50–200 nm) and thickness (10 to 200 nm) were found alongside cell-encapsulating meshes of EPS (Figures 3A1−C) (Lorite et al., 2011; Tu et al., 2020). Dohnalkova et al. (2011) performed in-depth comparative analysis of sample preparation for EM imaging of biofilm communities, where EPS was found to undergo structural characteristic changes depending on the means of sample drying. The EPS webbing and > 1 μm channels/ridged present in the HIM images are believed to be products of chemical processing (EtOH dehydration) and viscoelastic deformation brought on by the turbulent high-pressure environment necessary in CPD processing. To observe the biodegradation capabilities of the marine microbial taxa, colonizing cells and organic matter were chemically removed from 77-day incubated polyolefins. Under the dense biofilm layers and EPS webbing were evidence of biodegradation (e.g., cracks and pits; Figure 4). Development of irregular crevasses were observed on all three polymer types in contrast to unexposed controls. The occurrence of these features was more spatially abundant on LDPE, followed by HDPE and PP (LDPE > HDPE > PP).

### Polyolefin surface functional groups modification following marine incubation

Minute indications of biodegradation activities were observed on all polymer types. Samples were collected at each time point, cell debris removed and ATR-FTIR spectral analysis perform to characterize functional group changes present on substrate surface. By comparing the emergence and disappearance of peaks of the exposed plastics, natural UV and unexposed controls, changes in surface functional groups attributable to biodegradation were assessed (Supplementary Figures S3–S5). When analyzing the polyolefins, the presence of carbonyl (C=O), hydroxyl (O-H), methylene (-CH_2_-), and terminal (vinyl) double bonds (-C=CH_2_) functional groups were targeted based on their characteristic peaks, typically in the range of 1,850–1,650 cm^−1^, 3,700–3,000 cm^−1^, 1,650–1,600 cm^−1^, respectively, (Albertsson et al., 1987; Devi et al., 2019; Almond et al., 2020; Tu et al., 2020; Gao and Sun, 2021). Carbonyl and terminal double bond indexes were determined to account for polymer thickness differences (Albertsson et al., 1987; Hadar and Sivan, 2004; Tribedi and Dey, 2017; Almond et al., 2020). Whereas changes in percent crystallinity were assessed based on peak absorbance calculations described in Lanyi et al. (2018) and Tarani et al. (2023).

Post incubation, the largest average changes in functional groups were observed on LDPE at wavelengths of 2,000–600 cm^−1^ in accord with previous observations from *in-situ* degradation studies (Dey et al., 2020; Tu et al., 2020; Gao and Sun, 2021). When compared, all marine-incubated polymer samples possessed the C=O peak, although the peaks were consistently greater on LDPE and HDPE samples. Of all three polymers compared to their UV controls, the marine incubated PP samples were found to express the least functional group changes (Table 4; Supplementary Figures S3–S5). The emergence of a broad O-H peak (3,380 cm^−1^) and lesser carbonyl peaks (1,477 cm^−1^) were indications of non-UV hydrolysis by marine microbes forming hydroperoxide and hydroxyl groups on LDPE and HDPE. Paço et al. (2017) described the ability of a marine fungus (*Zalerion maritimum*) to degrade PE, as a product of the degradation distinct peaks at 3700–3000 cm^−1^ emerged and other reports (Tu et al., 2020). Specifically, the growth of the O-H peak (3,380 cm^−1^) dramatically increased 4-fold from day 56–70, which was similarly reported on day 75 and 135 marine incubated PE by Tu et al. (2020). However, unlike previous reports (Santo et al., 2013; Devi et al., 2019; Dey et al., 2020) which tested the biodegradation capability of the various bacterial isolates on PE, ANOVA tests revealed there were no statistically significant changes in carbonyl index values between the polymer treatments, with exception for the comparison of natural UV-LDPE to the unexposed LDPE (Table 4). However, LDPE exhibited a significant increase in percent crystallinity by day-77 (ANOVA: value of *p* = 0.006). The marine incubated and natural UV treatments when compared to unexposed controls exhibited a minute, yet significant difference (Tukey’s HSD test: *p*_adj_ = 0.048 and 0.005, respectively). An increase in percent crystallinity has been attributed to polymer reorganization following biodegradation via release of low molecular weight compounds from amorphous fractions (Sudhakar et al., 2007; Chamas et al., 2020).

**Table 4:** Carbonyl index, terminal double bond index and percent crystallinity of 77-day marine incubated polyolefin polymers in comparison to unexposed and natural UV exposed controls (*n* = 3).

Polymer	Treatment	Carbonyl index	Terminal double bond index	% Crystallinity
HDPE	Unexposed	0.156 ± 0.033	0.029 ± 0.002	72.8 ± 6.62
	Natural UV	0.091 ± 0.031	0.024 ± 0.01	80.2 ± 3.06
	Marine	0.156 ± 0.067	0.108 ± 0.053	78.9 ± 7.99
LDPE	Unexposed	0.408 ± 0.006	0.075 ± 0.016	61.5 ± 0.34
	Natural UV	0.506 ± 0.211	0.052 ± 0.021	69.5 ± 2.90**
	Marine	0.224 ± 0.054	0.159 ± 0.034*	66.4 ± 1.72*
PP	Unexposed	0.484 ± 0.079	0.142 ± 0.005	51.3 ± 1.40
	Natural UV	0.328 ± 0.134	0.177 ± 0.004*	50.0 ± 3.28
	Marine	0.254 ± 0.083	0.194 ± 0.016**	50.7 ± 0.71

Significance in comparison to unexposed controls: <0.001 [***], <0.01 [**], <0.05 [*].

Significant differences in the mean terminal double bond (-CH=CH_2_) index values of marine incubated LDPE and PP were observed (ANOVA; value of *p* = 0.0046 and 0.0016 respectively). Marine incubated treatments were significantly different from the unexposed controls (Tukey HSD: *p*_adj_ = 0.0158 and 0.0015). Development of terminal (vinyl) double bonds and reduction in carbonyl groups are indicative of biodegradation (Albertsson et al., 1987; Devi et al., 2019; Dey et al., 2020). The ATR-FTIR results related to the emergence of O-H groups, significant development of vinyl double bonds (LDPE/PP) and increase in crystallinity (LDPE), yet non-significant reduction of carbonyl groups suggests the exposed polyolefins were subject to a varying degree of biodegradation over the 77-day marine incubation.

## Discussion

### Polyolefin community composition in the oligotrophic waters of the Bermuda Platform

Numerous recent studies have investigated the composition of biofilms on polyolefins in marine environments, driven by interest in understanding microbial degradation of these environmental pollutants and potentially harnessing microbes to accelerate their breakdown (Sudhakar et al., 2007; Oberbeckmann et al., 2016; De Tender et al., 2017; Oberbeckmann et al., 2018; Dudek et al., 2020; Erni-Cassola et al., 2020; Pinto et al., 2020; Tu et al., 2020; Kumar et al., 2022; Latva et al., 2022; Wallbank et al., 2022). Through the application of 16S rDNA amplicon sequencing of the V4 region, we focused on rare members of colonizing communities, and changes in community composition over a lengthy incubation, postulating that some plastic-interacting taxa might grow slowly and appear only after a protracted period. Although by targeting the 16S rRNA V4 region, we must temper our reports of relative abundance regarding specific taxonomic groups that may be overrepresented (e.g., Nitrosopumilales) or underestimated (e.g., SAR11) as inconsistencies within the V4 primers have been documented (Apprill et al., 2015; Parada et al., 2016; Wear et al., 2018). Given that this investigation centers on the study of less abundant, slower growing taxa, we opted to invest resources into 16S rDNA gene amplicon based analyses, as it is better for the characterization of rarer taxa (Sharpton et al., 2011). In addition, 16S sequencing was implemented to facilitate interstudy meta-analyses of these types of communities, given that many prior plastisphere investigations have also targeted the V4 locus of the 16S gene (Jacquin et al., 2019; Roager and Sonnenschein, 2019; Wallbank et al., 2022). Unexpectedly, we observed a large transition in community composition 6–7 weeks into the 11-week incubation. The transition is associated with a sharp increase in alpha diversity observed across the material-types, where the pre-transition prominent taxonomic groups were replaced by a large variety of rare taxa. Within this set of rare taxa are previously characterized hydrocarbon utilizers and candidate plastic degraders that would be expected to grow slowly and only at the material surface.

The microbiome results from the first 6 weeks of our experiment were consistent with previous studies, ranging from the North Atlantic to the Caribbean (De Tender et al., 2017; Oberbeckmann et al., 2018; Wright et al., 2020). In our study, polyolefin-associated communities were dominated by Gammaproteobacteria, Alphaproteobacteria and Bacteroidia over the first 3 months of exposure. Prior research has shown that various bacterial families within these classes are common colonizers of polyolefins: Alcanivoracaeae, Comamonadaceae, Erythrobacteraceae, Flavobacteriaceae, Hyphomonadaceae, Oceanospirillaceae, Pseudoalteromonaceae, Rhodobacteraceae, Saprospiraceae, Sphingomonadaceae, and Vibrionaceae (Oberbeckmann et al., 2014; De Tender et al., 2015; Bryant et al., 2016; De Tender et al., 2017; Syranidou et al., 2017; Oberbeckmann et al., 2018; Devi et al., 2019; Kirstein et al., 2019; Oberbeckmann and Labrenz, 2019; Roager and Sonnenschein, 2019; Xu et al., 2019; Dey et al., 2020; Harvey et al., 2020). In our study, the dominant polyolefin colonizing families at varying timepoints were the Alteromonadaceae, Flavobacteriaceae, Marinomonadaceae, Rhodobacteraceae, Saccharospirillaceae, Saprospiraceae, Thalassospiraceae, Halieaceae and Vibrionaceae. When examined at the genus-level, the dominant genera were similar across polymer type and consistently showed declining abundance as the incubation progressed into the late period. The late period was characterized by a rapid increase in diversity across all material types. Where polyolefin communities were dominated by a myriad of rare genera (≤1% relative abundance), which combined abundance increased as time progressed: 66.4% (Day 56) to 92.9% (Day 77). Notable late period genera (>1%, in order of highest abundance) were *Saccharospirillum*, *Vibrio*, *Hyphomonas*, *Filomicrobium*, *Blastopirellula*, unclassified Rhodobacteraceae genus, Hyphomonadaceae, Micavibrionaceae, Saprospiraceae, Oligoflexaceae, Halieaceae and Cyclobacteraceae.

As observed by Syranidou et al. (2017), representatives of the Hyphomonadaceae and Marinomonadaceae were significantly more abundant on the polyolefins than the seawater communities. Significant differences between material-attached communities and seawater were expected and have been consistently reported (Elifantz et al., 2013; Oberbeckmann et al., 2016; Roager and Sonnenschein, 2019; Wright et al., 2020). However, whether there are significant differences between plastic communities and glass communities has been a topic of debate (Oberbeckmann et al., 2018). This subject of contention is rooted in the early plastisphere studies, where glass controls used in short duration *in-vitro* studies were found to be significantly different from their polymer counterparts (Parada et al., 2016; Wear et al., 2018). The results of this investigation and other *in situ* experiments support the growing consensus that there are no significant differences between polymer and glass colonizing communities when the most prominent taxa drive the analysis (Kirstein et al., 2016; Oberbeckmann et al., 2016; De Tender et al., 2017; Dudek et al., 2020). However, in support of the hypothesis that polyolefin interactions will impact a small subset of communities that interact slowly with the polyolefin surface, we observed significant differences in rare taxa between the various material-attached communities. The significant enrichment of some rare taxa on glass may indicate ecological selection driven by surface properties, production additives and/or the polyolefin colonizing communities selecting against them.

### A major shift in biofilm communities observed following Tropical Storm Henri

Between days 42 and 56 (August 5th and 19th, respectively), we observed an abrupt transition in material-attached communities. During that time, an environmental disturbance associated with the development of Tropical Storm Henri occurred from August 14th to 19th, 2021. Although the storm reached near gale winds (Max: 32 knots), changes in community composition of the ambient seawater (Figure 5B), and in seawater parameters (pH, temperature, conductivity, dissolved oxygen) were non-significant. We propose two alternative hypotheses that could explain observations from this uncontrolled experiment imposed by nature: [1] the transition might have been the result of the environmental disturbance, or [2] the biofilm communities may have been transitioning through a stage of community succession precipitated by processes unrelated to the hurricane.

Following the disturbance, the abrupt shift to the ‘late’ period can be characterized as a period of rapid diversification, where the number of highly abundant lineages decline, and a myriad of ‘rare’ taxa rise (Figures 5A,C). Of the few newly abundant lineages, the relative abundance of the Hyphomonadaceae, Rhodobacteraceae, Halieaceae and Saprospiraceae families increased. Saprospiraceae members (*Portibacter*, *Lewinella* and multiple unresolved genera) thrived and gradually increased in relative abundance throughout the late period. These taxa are important degraders of complex organic compounds, and the members that utilize helical gliding motility are commonly associated as predators of algal or bacterial cells (*Saprospira* spp., *Aureispira* spp. and some *Lewinella* spp.; McIlroy and Nielsen, 2014).

The tropical storm hypothesis can be rationalized by proposing the material-attached biofilm communities were altered by the hydraulic shear forces brought on by the tropical storm, where the degree of cellular disaggregation is dependent on the strength of the shear force (Liu and Tay, 2001). As similar, yet different changes in plastic associated communities have been documented following storm related disturbances. Nguyen et al. (2023) observed a significant change in plastisphere composition of marine incubated plastic netting following an intense storm in the Gulf of Aqaba. After the disturbance, the plastic net communities exhibited a sharp decrease in ɑ-diversity over the following 2 weeks, then an abrupt increase to greater than pre-disturbance levels over the span of 1 week. The difference between the species turnover of Nguyen et al. (2023) and this study may be attributable to the timing of our sampling or differences in the degree of biofilm cellular disaggregation. Where the environmental disturbance may have positively impacted the survival of rare colonizers eking out an existence on finite resources governed by competition and/or motile predators (e.g., *Saprospira-like* spp., *Bdellovibrio* spp.) that thrive in response to opportunistic predation on storm battered sessile community members (McIlroy and Nielsen, 2014). However, no significant indications of highly abundant predatory functions were observed in any of the polyolefin communities during the late period (Supplementary Figure S7).

The alternative hypothesis, which speculates that the transition was a natural consequence of community succession is supported by the observation that a similar transition was not observed in the ambient seawater community (Figure 5B). However, this could have been due to the suspended cells settling out of the surface layer by the time sampling occurred at day 56. Although it is plausible that the polyolefin communities would have undergone the observed successional changes in a progression towards a mature climax community, regardless of the tropical storm.

### Presence of HCBs and plastic-degradation potential

A few highly abundant hydrocarbonoclastic bacteria were found on all three polymer types throughout the incubation: *Alteromonas*, *Marinomonas*, *Oleibacter* and *Hyphomonas* (Albertsson et al., 1987; Yakimov et al., 2007; Dohnalkova et al., 2011; Hirai et al., 2011; Lorite et al., 2011; Hahladakis et al., 2018; Kirstein et al., 2019; Yakimov et al., 2019; Semenova et al., 2022; Yakimov et al., 2022). These taxa are known to possess alkane-1-monooxygenase genes, an enzyme required for hydrocarbon/n-alkane degradation (Semenova et al., 2022). The order Oceanospirillales includes a wide array of hydrocarbon utilizers (predominantly polyaromatic hydrocarbon (PAH) degradation), most notably Oceanospirillaceae (*Oleibacter* spp.*, Marinomonas* spp.), Halomonadaceae (*Halomonas* spp.), and Alcanivoraceae (*Alcanivorax* spp., *Ketobacter* spp.; Teramoto et al., 2011; Dong et al., 2015). Also present were prosthecate bacteria; Hyphomonadaceae members that produce polysaccharide holdfasts to attach securely to surfaces. Some taxa within the Hyphomonadaceae have been described as putative hydrocarbon degraders of oil contaminated sites, are known to be common colonizers of polyolefins and have been isolated from petroleum contaminated sites (Nie et al., 2014; Oberbeckmann and Labrenz, 2019). The Oceanospirillales members *Thalassospira* spp. and *Alteromonas* spp. commonly colonize a myriad of plastic polymers and are hypothesized to carry out a range of niche roles within the plastisphere community. These roles have been hypothesized to include generalist hydrocarbon degradation, which on the surface of plastic polymers could be related to the uptake of phytoplankton hydrocarbon products produced early in biofilm formation, and/or the degradation of plastic-associated alkane dimers/monomers (Chronopoulou et al., 2015; Amaral-Zettler et al., 2020; Cheng et al., 2021).

It has been suggested that if an organism degrades the substrate it interacts with, the abundance of that organism should not be rare (Debroas et al., 2017). However, this statement does not account for competition for exposed polymer surfaces, or for the extremely low rates of polyolefin degradation that have been observed in natural environments. The HIM images of the polyolefin colonized surfaces (Figure 2) and artifacts of biodegradation (Figure 4; Table 4) portray a community in which select marine taxa may perform plastic degradation on exposed sites. When exploring microbial associations on plastic polymers, multiple studies have documented a variety of significant polymer-microbe enrichments (Zettler et al., 2013; Debroas et al., 2017; Syranidou et al., 2017; Oberbeckmann et al., 2018; Roager and Sonnenschein, 2019; Erni-Cassola et al., 2020; Latva et al., 2022). Debroas et al. (2017), found OTUs of *Thalassospira* spp. alongside 5 other punitive polyester degraders significantly associated with various plastic waste (PE, PS and PET). Oberbeckmann et al. (2018) and Zettler et al. (2013) observed enriched abundances of Erythrobacteraceae (*Erythrobacter* sp.) and Hyphomonadaceae (*Hyphomonas* sp.) genera on PE over seawater and natural substrates. Hydrocarbon utilization activities on a range of organic hydrocarbons (PAHs to n-alkanes) have been characterized for many of these enriched taxa (Yakimov et al., 2007, 2019, 2022; Dong et al., 2015). Here, we report the first consistent significant enrichment of a *Ketobacter* member on LDPE throughout an *in situ* experiment. *Ketobacter* spp. have previously been associated as highly specialized degraders of linear and branched alkanes (Yakimov et al., 2019; Sun et al., 2022). As biofilm formers and biosurfactant producers, Alcanivoracaceae representatives (*Ketobacter* spp. and *Alcanivorax* spp.) are efficient degraders of C_9_–C_30_ alkanes and have a high affinity for LDPE colonization (Head et al., 2006; Yakimov et al., 2019; Pinto et al., 2022). However, LDPE degradation capabilities have yet to be assessed.

### Biofilm formation and compositional changes of the polyolefin plastisphere

Biofilm formation and development times can be hampered or expedited depending on the nutrient availability in the marine environment. Various bacterial species have been observed to transition from planktonic to surface-attached lifestyles given low-nutrient conditions. Whereas in nutrient-rich conditions, the taxa may express higher preference to be free-living (Stanley and Lazazzera, 2004). However, this is not ubiquitous among all bacteria, it is inferred that in nutrient-rich environments, biofilm development is rapid due to the abundance of available nutrients which enables quicker development of various colonization stages (Datta et al., 2016; Oberbeckmann et al., 2018). Currently, there is no agreement on distinct biofilm formation rate on plastics, however various environmental factors have been implicated to influence it.

While observing biofilm formation on PE in the Yellow Sea, Tu et al. (2020) reported incubation time, but not depth to be significant. Shifts in community composition occurred in 3 phases: ‘Early’ phase (day 30), mid-shift (day 75) and late shift (day 135). De Tender et al. (2017) found that sample type (PE sheet, dolly rope, seawater, or sediment), environment (harbor v. offshore) and exposure time (0–44 weeks) significantly affected the biofilm composition. As previously mentioned, Nguyen et al. (2023) observed a significant change in community composition following an environmental disturbance after an incubation period of 9 days. Alongside these factors, spatial location, substrate type and size, seawater temperature, oxygen content, light, pH, nutrient availability, and salinity have been associated as significant influencing factors in plastic colonization and biofilm formation (Oberbeckmann et al., 2014; De Tender et al., 2017; Kirstein et al., 2019; Xu et al., 2019; Tu et al., 2020; Wright et al., 2020; Rummel et al., 2021). Over the exposure period, we observed a gradual decline in Gammaproteobacteria and increase in Alphaproteobacteria and Bacteroidia like De Tender et al. (2017). However, unlike Tu et al. (2020), we observed a single stark transition in community composition expressed on all plastic and glass control samples following an environmental disturbance (between days 42 and 56). Post transition, many of the prominent colonizing taxa sharply declined and were replaced by a swath of generalist hydrocarbon degraders (e.g., Hyphomonadaceae and Rhodobacteraceae) and predatory bacteria (e.g., Saprospiraceae and Micavibrionaceae). Our findings support the notion that formation/succession rates are not finite and are highly influenced by the environment the plastics are exposed to.

### Polyolefin community interactions

Within biofilm communities there are dynamic interactions between individual cells and their microbial neighbors. These ecosystem interactions vary and result in different outcomes depending on the type of interaction: competition, predation, or symbiosis (mutualism, commensalism, parasitism). The insight into the metabolic and ecological functions described in this report should not be seen as absolute. The labeled functions are not based on the analysis of specific genes sourced from metagenomic data, but instead functions sourced from the Faprotax database and assigned based on 16S rDNA sequence taxonomy (Louca et al., 2016). As such, the community functional analysis may not account for the functions associated with novel or uncultured taxa as only 68–78% of the late period functions were annotated on average.

With that said, the most abundant functions present within the general polyolefin-colonizing communities (8,641 ASVs) were fermentation and nitrite respiration by far. Those functions were accompanied by cellulolysis and multiple other functions that minutely increased as time progress (Supplementary Figure S7). The acquisition of carbon by members of the plastisphere is diverse, ranging from various forms of phototrophy to heterotrophy. It been reported that some heterotrophic colonizers utilize metabolites of co-colonizers like phytoplankton photosynthates or actively prey upon other community members (Zettler et al., 2013; Mishamandani et al., 2016; Dussud et al., 2018; Cheng et al., 2021). When observing the rare community members associated with the 594 significantly enriched ASV group, the most abundant functions were aerobic chemoheterotrophy followed by fermentation, intracellular parasites, nitrate reduction, intracellular parasites, cyanobacterial phototrophy, predatory/exoparasitic, and nitrate respiration (Supplementary Figure S8).

When probing for hydrocarbon degradation functions among the general polyolefin colonizers their relative abundance was nearly undetectable. However, within the rare significantly enriched ASVs, hydrocarbon degradation (e.g., broad range hydrocarbon degradation and aliphatic non-methane hydrocarbon degradation) minutely grew in abundance representing ~0.5–2% of the community functions as time progressed following the mid-experimental transition. This aligns with the assumption that if plastic degradation was to occur, it would be performed by rare slow growing community members. Although only a miniscule fraction of the overall community was annotated with hydrocarbon degradation, a metagenome-based functional investigation is necessary to extract more extensive information related to plastic degradation capabilities of polyolefin colonizers of the Bermuda Platform.

Because complete depolymerization and remineralization of polyolefin plastic is an energetically expensive and unfavorable process for a single community member to perform, it is often assumed that commensal and/or mutualistic interactions of bacterial consortia result in plastic biodegradation. However, biodegradation by a single species is possible, as in the case of *Ideonella sakaiensis,* a soil bacterium capable of PET degradation using a two-enzyme system (Yoshida et al., 2016). Although, our data suggests that plastic biodegradation in natural marine environments is slow and might occur in response to the collaborative effort of multiple microbial taxa. The degradative actions by few taxa specialists may subsequently enable depolymerization activities by a range of hydrocarbon generalists. These degradative activities may also occur as the result of inter-kingdom community interactions between bacterial and fungal colonizers (De Tender et al., 2017).

## Conclusion

This study was conducted to observe the microbial community colonization and succession on polyolefins in a coastal oligotrophic marine system over 3-months and identify significant polymer-associated taxa. Over the experimental period, early polyolefin colonization (<1 month) was characterized by high relative abundance of few genera, specifically *Marinomonas*, *Thalassospira*, *Vibrio*, *Oleibacter* and *Alteromonas*. These genera were found to sharply decline following an apparent successional event observed between days 42 and 56, in which a pronounced increase in biofilm diversity was observed. When compared between substrate types, the composition of plastic and glass communities were significantly different compared to the seawater community, but not between the plastic types and/or glass, except in the case of rare microbiota. Differential abundance analyses identified 594 of 8,641 colonizing taxa were significantly enriched on one or more polymer types at various incubation times, and furthermore, 25 possessed significant enrichment on one polymer type throughout the entire incubation. When assessing influential factors, incubation time was the only factor found to exert a significant influence on abundant taxa in colonizing communities throughout the experiment. However, polymer type influenced the rare community membership, particularly after the rapid successional transition following day 42.

Polyolefin residence in a surface marine system did not result in significant quantitative evidence of biodegradation. Although, minute evidence of polymer functional group and surface modifications were observed. Here, we report evidence of multiple microbial taxa that were enriched on specific plastic types in long-term incubations of plastics in marine environment. Some of these taxa were not previously known to be associated with plastics. These taxa may represent new plastics degrading types that were uncovered by our experiments because of the incubation time length and our focus on rare taxa that may represent plastic degraders found deep in biofilms at the plastics surface. These organisms should be viewed as potential targets for future investigations, singly and in consortia, aimed at understanding and simulating plastic degradation in the marine environment. Our prospective research ventures aim at the retrieving significant substrate enriched isolates from the cultures yielded to screen for polyolefin degradation capabilities.

## Data availability statement

The datasets presented in this study can be found in online repositories. The names of the repository/repositories and accession number(s) can be found at: NCBI - PRJNA1005706.

## Author contributions

SS: Conceptualization, Data curation, Formal analysis, Investigation, Methodology, Project administration, Software, Validation, Visualization, Writing – original draft, Writing – review & editing. ED: Data curation, Formal analysis, Software, Validation, Visualization, Writing – review & editing. HA: Formal analysis, Software, Validation, Visualization, Writing – review & editing. AD: Writing – review & editing, Investigation. SB: Methodology, Resources, Writing – review & editing. RP: Writing – review & editing, Methodology, Funding acquisition, Resources. TS: Conceptualization, Funding acquisition, Methodology, Project administration, Resources, Supervision, Writing – review & editing. SG: Conceptualization, Funding acquisition, Methodology, Project administration, Resources, Supervision, Writing – review & editing.
