# Supplementary material for: Identification of rare microbial colonizers of plastic materials incubated in a coral reef environment
Source: Front Microbiol. 2023 Oct 5;14:1259014. doi: 10.3389/fmicb.2023.1259014 (PMC10585116; doi:10.3389/fmicb.2023.1259014)
Supplement: Supplementary file 1 [file Data_Sheet_1.pdf]

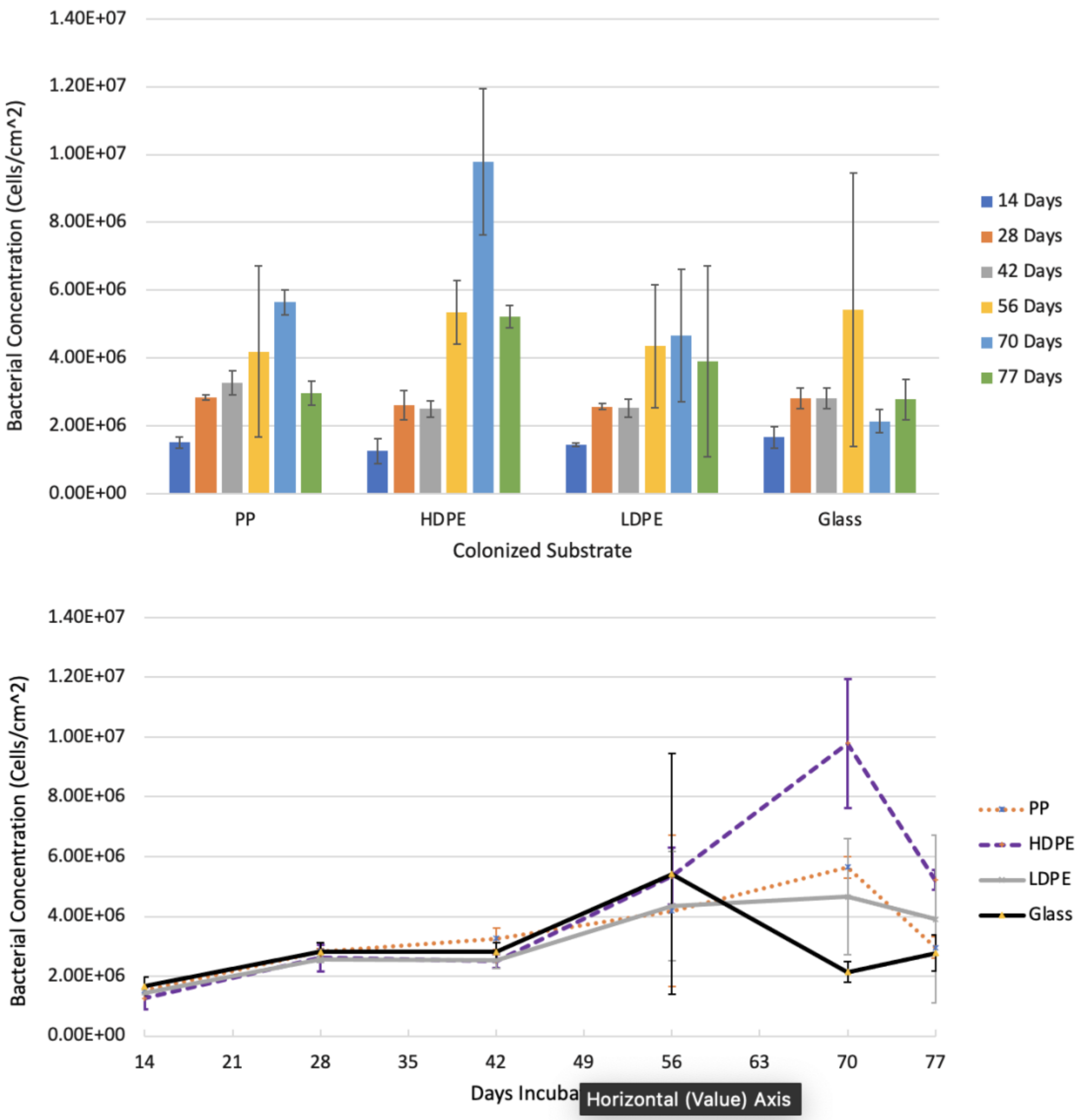

Supplementary Figure 1. Change in material-colonized bacterial biomass over 11 weeks. Bar charts of DAPI-stained cell enumeration values for each plastic type and glass control over the entire incubation period. The bacterial concentrations reported are the averaged counts per cm2 for each material type (n = 3) and error bars represent within sample standard deviation. All cell counts were collected using an Olympus Provis florescent microscope.

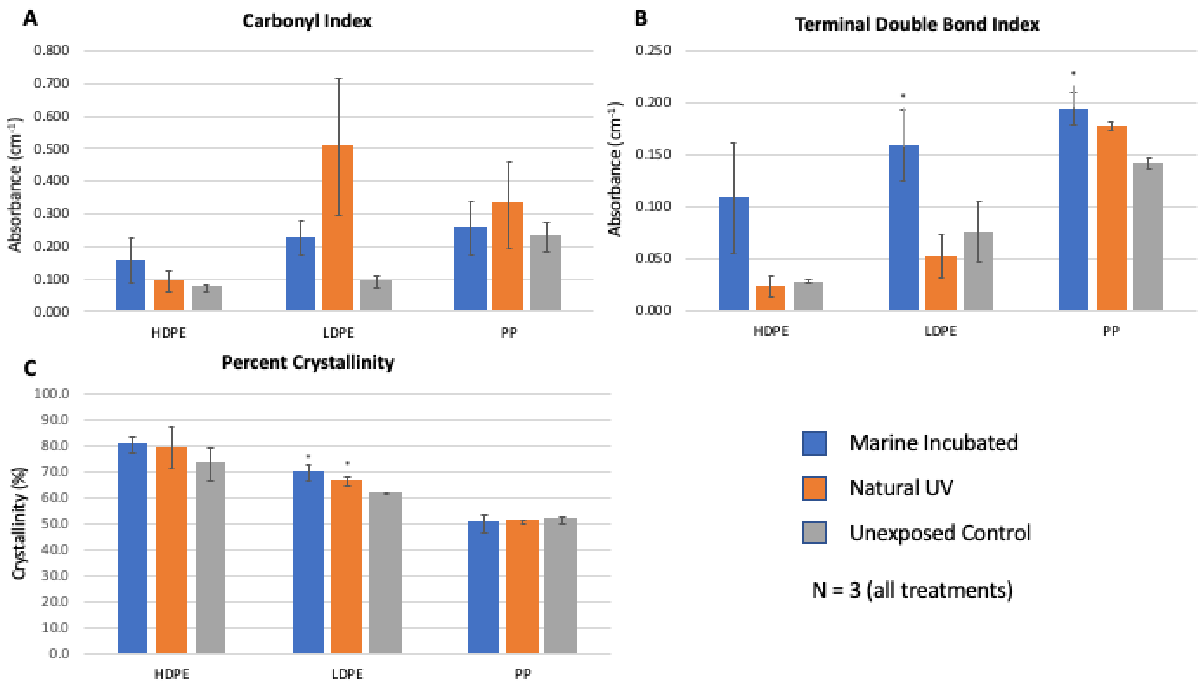

Supplementary Figure 2. Bar charts of (A) carbonyl index, (B) terminal (vinyl) double bond index and (C) percent crystallinity for each polymer type and treatment. Blue: 77-day surface seawater incubated polyolefins; Orange: 77-day natural UV exposed polyolefins; Grey: Unexposed, untreated polyolefins. Asterisks denote statistical significance (ANOVA; * = pvalue <0.05, ** < 0.01) compared to unexposed controls.

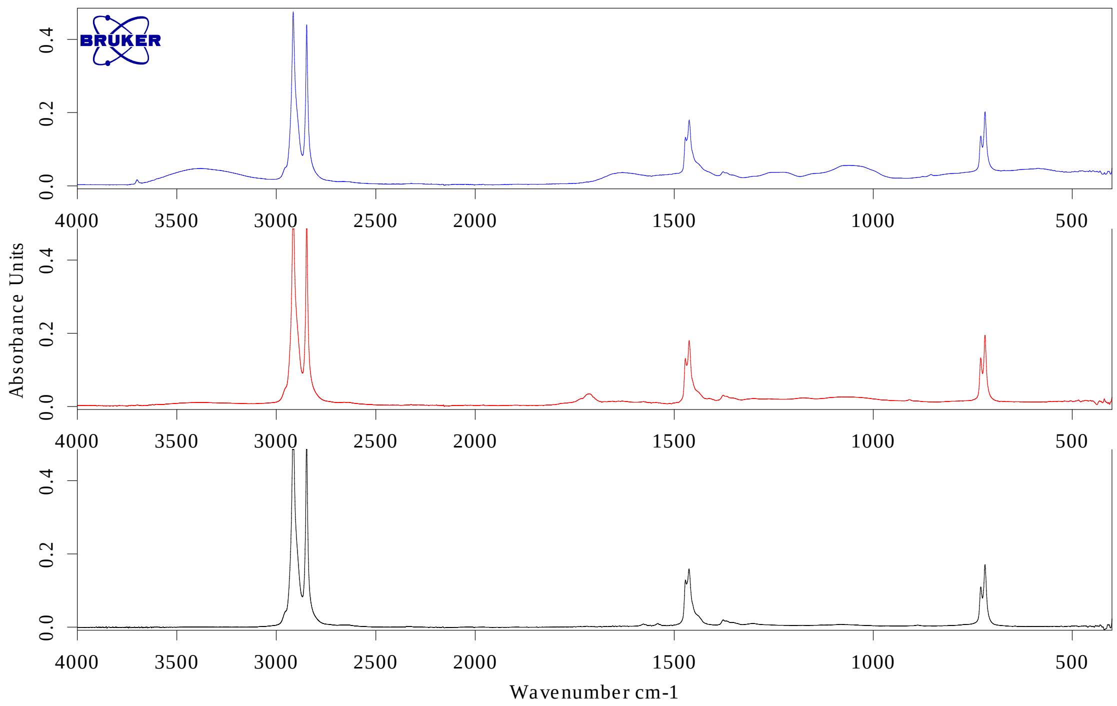

C:\Program Files (x86)\OPUS\WORK\Av.1 Average of Day 77 LDPE Marine Incubated Samples		2021/10/15
C:\Program Files (x86)\OPUS\WORK\Av.0 Average of Day 77 LDPE Abiotic Controls		2021/10/22
C:\Program Files (x86)\OPUS\WORK\Av.2 Average of Day 77 LDPE Virgin Controls		2021/4/22

Supplementary Figure 3. Comparative stacked absorbance plots of ATR-FTIR measured LDPE spectra. The stacked peak absorbance plots showcase a wide array of differences in the emergence of various functional group peaks between three LDPE treatments: 77-day marine incubated (BLUE), 77-day natural UV control (RED), and 77 unexposed virgin control (BLACK). Formation of a broad hydroxyl (-OH) peak between 3700-3000 cm-1, a broad ether (C-O-C) / hydroxyl peak between 1100-1000 cm-1, a small alkene (C=C) peak 1640-1630 cm-1, and the reduction/formation of carbonyl functional groups between 1740-1700 cm-1 in contrast to the UV and Virgin controls.

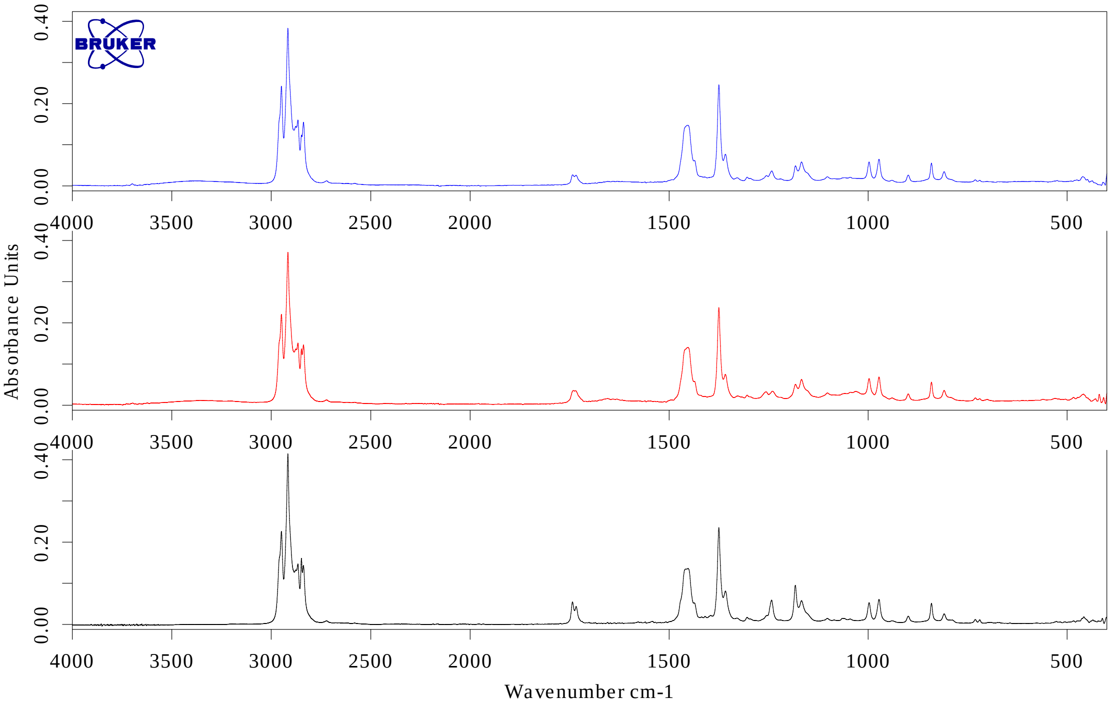

C:\Program Files (x86)\OPUS\WORK\Av.6	Average of Day 77 PP Marine Incubated Samples	2021/10/15
C:\Program Files (x86)\OPUS\WORK\Av.7	Average of Day 77 PP Abiotic Controls	2021/10/22
C:\Program Files (x86)\OPUS\WORK\Av.8	Average of Day 77 PP Virgin Controls	2021/4/22

Supplementary Figure 4. Comparative stacked absorbance plots of ATR-FTIR measured HDPE spectra. The stacked peak absorbance plots showcase the difference in the emergence of various functional group peaks between three HDPE treatments: 77-day marine incubated (BLUE), 77-day natural UV control (RED), and 77 unexposed virgin control (BLACK). Important differences that were expressed on the marine incubated samples was the formation of a broad hydroxyl (-OH) peak between 3700-3000 cm-1and a slight alkene (C=C) peak between 1640-1630 cm-1, in contrast to the UV and Virgin controls.

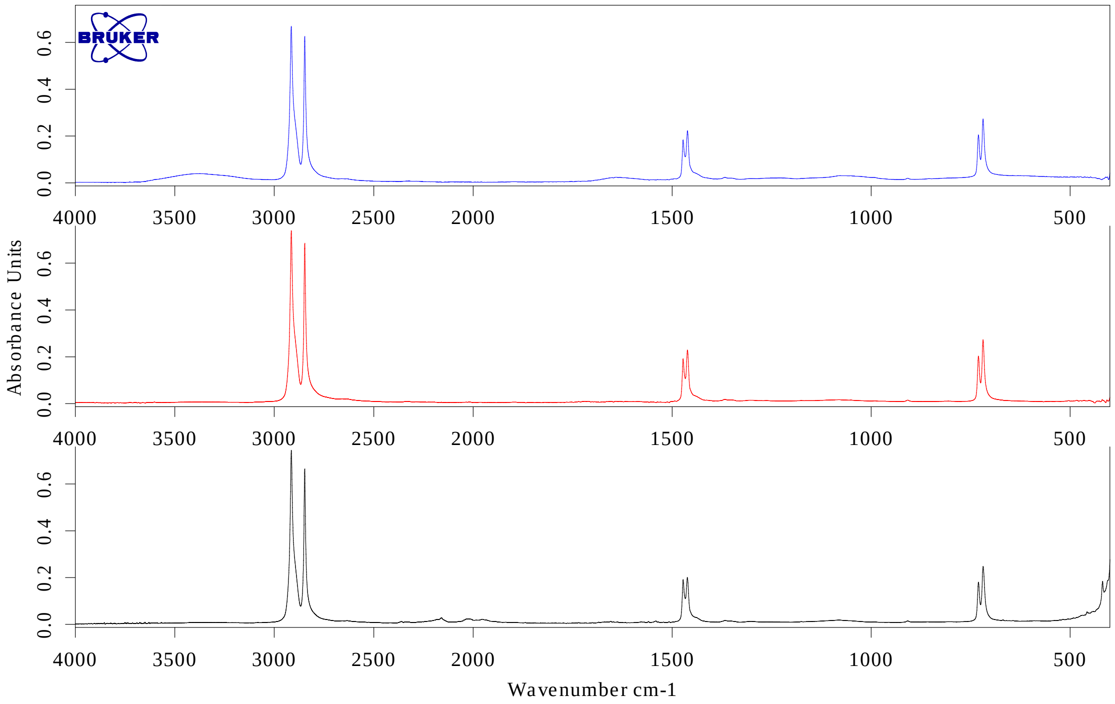

C:\Program Files (x86)\OPUS\WORK\Av.9	Average of Day 77 HDPE Marine Incubated Samples	2021/10/15
C:\Program Files (x86)\OPUS\WORK\Av.4	Average of Day 77 HDPE Abiotic Controls	2021/10/22
C:\Program Files (x86)\OPUS\WORK\Av.5	Average of Day 77 HDPE Virgin Controls	2021/4/22

Supplementary Figure 5. Comparative stacked absorbance plots of ATR-FTIR measured PP spectra. The stacked peak absorbance plots showcase little to no difference in the emergence of various functional group peaks between three PP treatments: 77-day marine incubated (BLUE), 77-day natural UV control (RED), and 77 unexposed virgin control (BLACK). Minute differences were expressed on the marine incubated samples which include the slight formation of a broad hydroxyl (-OH) peak between 3700-3000 cm-1 and the slight reduction of carbonyl functional groups between 1740-1700 cm-1 in contrast to the UV and Virgin controls.

ASV	Kingdom	Phylum	Class	Order	Family	Genus
Ref1	Bacteria	Proteobacteria	Alphaproteobacteria	Rhodobacterales	Rhodobacteraceae	Albimonas
Ref2	Bacteria	Proteobacteria	Alphaproteobacteria	Rhodobacterales	Rhodobacteraceae	Albimonas
Ref3	Bacteria	Proteobacteria	Alphaproteobacteria	Rhodobacterales	Rhodobacteraceae	Oceanicella
Ref4	Bacteria	Proteobacteria	Alphaproteobacteria	Rhodobacterales	Rhodobacteraceae	Oceanicella
ASV1	Bacteria	NA	NA	NA	NA	NA
ASV2	Bacteria	NA	NA	NA	NA	NA

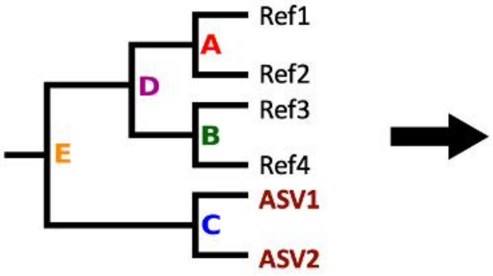

1. Label internal nodes with known reference labels
A: Rhodobacteraceae *Albimonas*
B: Rhodobacteraceae *Oceanicella*
D: Rhodobacteraceae
E: Rhodobacteraceae

2. Propagate internal node taxonomic labels to unknown ASVs
C: Rhodobacteraceae
ASV1: Rhodobacteraceae
ASV2: Rhodobacteraceae

Supplementary Figure 6. Cladal Taxonomic Annotations: ASVs and full-length sequences are aligned and combined into a phylogenetic tree. Each internal node is assigned with the most specific Linnean taxonomic label that is shared by all descendants within that clade. For example, Clade A above can be assigned to genus level while Clade D only shares taxonomic labels among all the descendants at Family level. Following labeling all internal nodes with information of reference taxonomic labels, unknown ASVs are labeled by propagating information from the most recent internal ancestor node which contains taxonomic labeling assigned from reference sequences. In this case, Clade E is the most recent common ancestor with known reference taxonomic annotations, and so both ASVs in this clade are assigned to family Rhodobacteraceae.

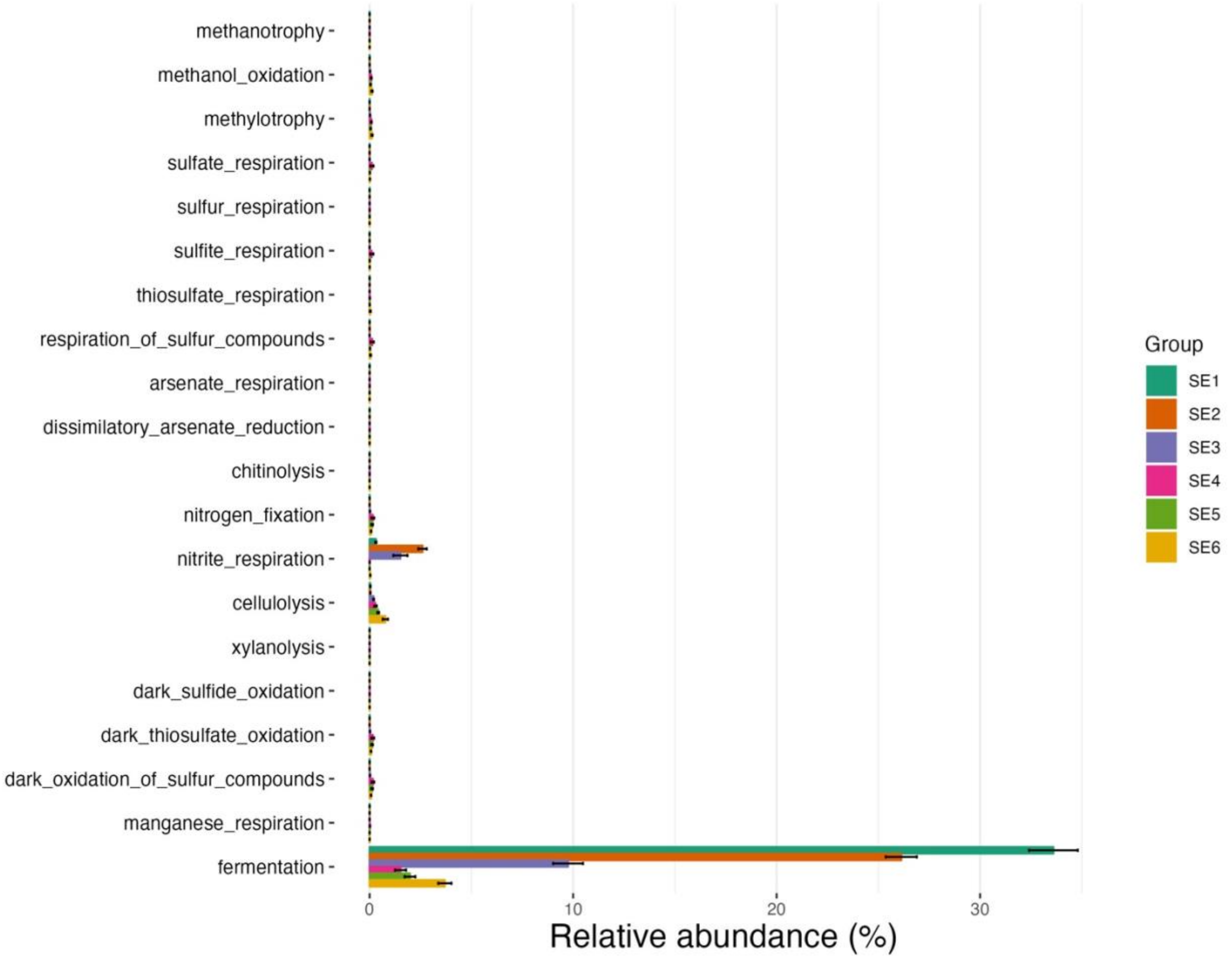

Supplementary Figure 7. Bar chart of community functions of all 8,641 bacterial polyolefin colonizing ASVs over time. Shown are the top 20 abundant metabolic and ecological functions present within all three polyolefin polymer communities.

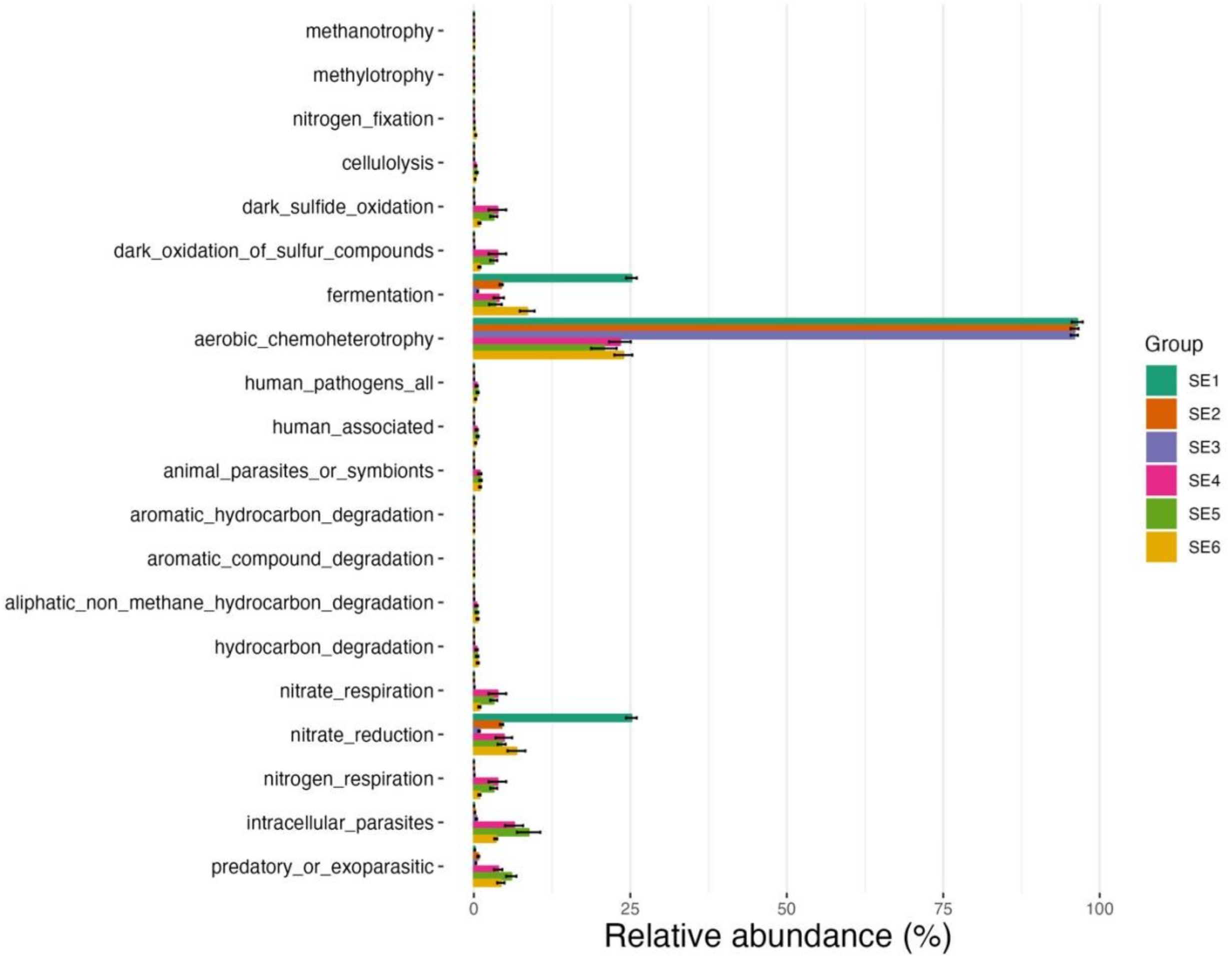

Supplementary Figure 8. The bar chart displays the differential abundance of the top 20 community functions of all 594 periodically significantly polyolefin enriched ASVs over time.
